# An advanced sheep (*Ovis aries*, 2*n* = 54) cytogenetic map and assignment of 88 new autosomal loci by fluorescence *in situ* hybridization and R-banding

**DOI:** 10.1111/j.1365-2052.2007.01598.x

**Published:** 2007-06-01

**Authors:** G P Di Meo, A Perucatti, S Floriot, H Hayes, L Schibler, R Rullo, D Incarnato, L Ferretti, N Cockett, E Cribiu, J L Williams, A Eggen, L Iannuzzi

**Affiliations:** * Laboratory of Animal Cytogenetics and Gene Mapping, National Research Council (CNR), ISPAAM Naples, Italy; † INRA, Laboratory of Biochemical Genetics and Cytogenetics, Jouy-en-Josas France; ‡ Department of Genetics and Microbiology, University of Pavia Pavia, Italy; § Department of Animal, Dairy, and Veterinary Sciences, Utah State University Logan, UT, USA; ¶ Parco Tecnologico Padano, Polo Universitario, Via Einstein Lodi, Italy

**Keywords:** cytogenetic map, FISH-mapping, gene, nomenclature, sheep

## Abstract

Presented herein is an updated sheep cytogenetic map that contains 452 loci (291 type I and 161 type II) assigned to specific chromosome bands or regions on standard R-banded ideograms. This map, which significantly extends our knowledge of the physical organization of the ovine genome, includes new assignments for 88 autosomal loci, including 74 type I loci (known genes) and 14 type II loci (SSRs/microsatellite marker/STSs), by FISH-mapping and R-banding. Comparison of the ovine map to the cattle and goat cytogenetic maps showed that common loci were located within homologous chromosomes and chromosome bands, confirming the high level of conservation of autosomes among ruminant species. Eleven loci that were FISH-mapped in sheep (*B3GAT2, ASCC3, RARSL, BRD2, POLR1C, PPP2R5D, TNRC5, BAT2, BAT4, CDC5L* and *OLA-DRA*) are unassigned in cattle and goat. Eleven other loci (*D3S32, D1S86, BMS2621, SFXN5, D5S3, D5S68, CSKB1, D7S49, D9S15, D9S55* and *D29S35*) were assigned to specific ovine chromosome (OAR) bands but have only been assigned to chromosomes in cattle and goat.

## Introduction

Cytogenetic maps, available for several domestic ruminants, are useful tools for studying complex animal genomes and chromosome evolution among bovid species (Piumi *et al.* 1998; Robinson *et al.* 1998; Di Meo *et al.* 2000; 2002; 2005; Iannuzzi *et al.* 2000a,b; 2001a) and between bovid species and humans (Schibler *et al.* 1998a; Di Meo *et al.* 2000; 2002; 2006; Iannuzzi *et al.* 2000b; 2001a). Assignment of individual genes and markers to the physical map allows the identification of rearrangements within conserved chromosome segments that have been designated using chromosome painting probes (Hayes 1995; Iannuzzi *et al.* 1998; 1999), as well as defines the complex rearrangements that differentiate humans and bovids. Other practical applications of these maps are in clinical cytogenetics, to better define the chromosomal rearrangements and abnormalities that may be involved in abnormal phenotypes (Iannuzzi *et al.* 2001b; Pinton *et al.* 2005). Cytogenetic maps are also essential for anchoring linkage and RH maps to specific chromosome regions and to define the order and orientation of linkage groups for which there is poor evidence from the RH and linkage mapping data (Gautier *et al.* 2002).

Although many loci have been assigned to cattle, sheep and goat genomes by linkage and RH mapping, a relatively small percentage of loci have been physically located to single chromosomal regions or bands. Unfortunately, very few studies have used both RH and FISH data for confirmation of RH-map construction across whole chromosomes or specific regions (Gautier *et al.* 2002).

The only cytogenetic map for sheep is available through SheepBase (http://www.thearkdb.org/species.html). This map includes a few well-positioned markers, but uses an old ideogram that differs from that reported in the latest standard chromosome nomenclature (ISCNDB 2001). A more detailed cytogenetic map covering all chromosome regions and constructed on the basis of the latest international chromosome nomenclature (ISCNDB 2001) is still lacking in this very important species.

In this study, a new and advanced cytogenetic map of sheep that contains 452 loci and covers almost all of the chromosome bands (mainly R bands) is presented. The map uses published data and the latest standard chromosome nomenclature (ISCNDB 2001). The map includes 88 loci, including 74 type I loci (known genes) and 14 type II loci (SSRs/microsatellite marker/STSs), assigned by FISH and R-banding for the first time in sheep.

## Materials and methods

Synchronized peripheral blood cell cultures and slide preparation steps were carried out as reported earlier (Di Meo *et al.* 2005). Caprine BACs containing type I and type II loci were identified by PCR screening of the INRA goat BAC library (Schibler *et al.* 1998a) and have been previously used to build comparative maps between ruminants, pig, horse and humans (Schibler *et al.* 1998b; Di Meo *et al.* 2000; 2002; 2006; Iannuzzi *et al.* 2000b; 2001a; Pinton *et al.* 2000; Milenkovic *et al.* 2002; Hayes *et al.* 2003). Likewise, bovine BAC clones containing type I and type II loci were identified after PCR screening of the INRA bovine BAC library with appropriate primers as described by Eggen *et al.* (2001). This BAC library was used to construct a first draft of a physical map of the bovine genome and over 26 000 BAC clones of the library were end-sequenced and are thus available as BES (BAC-end sequences) in GenBank (Schibler *et al.* 2004; 2006). Cattle and goat BAC libraries are available to the entire research community through the GADIE Biological Resources Center (http://www-crb.jouy.inra.fr/BRC/index.html). Table 1 summarizes information about all BAC probes used for this study.

**Table 1:** Autosomal loci mapped by FISH to ovine (OAR) chromosomal locations.

Locus symbol	Locus name	Clone	OAR	BTA	HSA
*D3S32 (ILSTS096)*	*DNA segment*	0337C07^1^	1p13	3q	————-
*TCHH* (previous alias: *THH*)	*trichohyalin*	240D1^2^	1p21	3q21	1q21-q23
*CDC20*	*CDC20 cell division cycle 20 homolog (*Saccharomyces cerevisiae*)*	13A6^2^	1p35	3q35	1p34.1
*CCT8*	*chaperonin containing TCP1, subunit 8 (theta)*	325A10+ 802F11^1^	1q12.2	1q12.2	21q21.3–21q22.1
*CASR*	*calcium-sensing receptor (hypocalciuric hypercalcaemia 1, severe neonatal hyperparathyroidism)*	139D12^1^	1q31	1q31	3q21-q24
*UMPS*	*uridine monophosphate synthetase (orotate phosphoribosyl transferase and orotidine-5*′-decarboxylase)	296A8^2^	1q31	1q31	3q13
*AGTR1*	*angiotensin II receptor, type 1*	361C8^2^	1q41dist	1q42	3q21-q25
*GYG1* (previous alias:*GYG*)	*glycogenin 1*	399A5^2^	1q41dist	1q42	3q24-q25.1
*TFDP2*	*transcription factor Dp-2 (E2F dimerization partner 2)*	290G12^2^	1q43	1q43prox	3q23
*TF*	*transferrin*	163H4^2^	1q43dist	1q43dist	3q21
*COL6A1*	*collagen, type VI, alpha 1*	0133E09^1^	1q45	1q45	21q22.3
*D1S86 (BMS922)*	*DNA segment*	0866A05^1^	1q45	1q	————-
*GALT*	*galactose-1-phosphate uridylyltransferase*	112G9^2^	2p13	8q13	9p13
*VLDLR*	*very low density lipoprotein receptor*	349B11^2^	2p17	8q17	9p24
*SFTPC*	*surfactant, pulmonary-associated protein C*	0533D05^1^	2p23prox	8q21dist	8p21
*GSN*	*gelsolin (amyloidosis, Finnish type)*	253E10^2^	2p27dist	8q28	9q33
*EN1*	*engrailed homolog 1*	438B7^2^	2q33	2q33	2q13-q21
*SLC11A1* (old alias: *NRAMP1*)	*solute carrier family 11 (proton-coupled divalent metal ion transporters), member 1*	264F4^2^	2q43	2q43	2q35
*PAX3*	*paired box gene 3 (Waardenburg syndrome 1)*	337A3^2^	2q43	2q43	2q35-q37
*TMEM50A* (old alias: *SMP1*)	*transmembrane protein 50A*	262H6+ 284H5^2^	2q45	2q45prox	1p36.11
*TGFA*	*transforming growth factor, alpha*	211C11^2^	3p14	11q14	2p13
*BMS2621*	*DNAs segment*	372H08^1^	3p14	11q	————-
*SFXN5*	*sideroflexin 5*	0904C05^1^	3p14	11	2p13
*POMC*	*proopiomelanocortin (adrenocorticotropin/beta-lipotropin/ alpha-melanocyte stimulating hormone/beta-melanocyte stimulating hormone/beta-endorphin)*	503E3^2^	3p24	11q24dist	2p23
*D5S3 (ETH10)*	*DNA segment*	0356G02^1^	3q21prox	5q	————-
*D5S68 (BMS1658)*	*DNA segment*	0771B05^1^	3q33	5q	————-
*HGF*	*hepatocyte growth factor (hepapoietin A; scatter factor)*	217E9^2^	4q22prox	4q15dist-21	7q21.1
*NPY*	*neuropeptide Y*	342C1^2^	4q26 prox	4q25-q26	7p15.3
*SSBP1*	*single-stranded DNA binding protein 1*	264C10^2^	4q34 dist	4q34dist	7q34
*VAV1*	*vav 1 oncogene*	72E3^2^	5q15prox	7q15prox	19p13.2
*GM2A*	*GM2 ganglioside activator*	336E12^2^	5q15	7q21	5
*HSPA4*	*heat shock 70 kDa protein 4*	544F11^1^	5q22.1	7q22.1	5q31.1-q31.2
*CSKB071*	*DNA segment*	0078E01^1^	5q22.1	7q	————-
*D7S49 (BMS792-D0S246)*	*DNA segment*	0478C12^1^	5q22.3	7q	————-
*IL12B*	*interleukin 12B (natural killer cell stimulatory factor 2, cytotoxic lymphocyte maturation factor 2, p40)*	0006B03^1^	5q24	7q23-q24	5q31.1-q33.1
*HADH* (old alias:*HADHSC*)	*L-3-hydroxyacyl-Coenzyme A dehydrogenase, short chain*	232A9^2^	6q15prox	6q15prox	4q22-q26
*D6S29 (IDVGA65)*	*DNA segment*	0980A05^1^	6q17	6q22	————-
*HMGCR*	*3-hydroxy-3-methylglutaryl- Coenzyme A reductase*	39C1^2^	7q13prox	10q12	5q13.3-q14
*MYH7*	*myosin, heavy polypeptide 7, cardiac muscle, beta*	86E2^2^	7q15	10q15-q21	14q11.2-q13
*MGAT2*	*mannosyl (alpha-1,6-)-glycoprotein beta-1,2-*N*-acetylglucosaminyl- transferase*	359E10^2^	7q24	10q24	14q21
*SORD*	*sorbitol dehydrogenase*	201F1^2^	7q32	10q32	15q15-q21.1
*SPTB*	*spectrin, beta, erythrocytic (includes spherocytosis, clinical type I)*	194B4^2^	7q34prox	10q34prox	14q24.1-q24.2
*TGM1*	*transglutaminase 1 (K polypeptide epidermal type I, protein-glutamine- gamma-glutamyltransferase)*	265C8^2^	7q34	10q34	14q11.2
*TGFB3*	*transforming growth factor, beta 3*	161F12^2^	7q34dist	10q34dist	14q24
*D9S15 (BM2504)*	*DNA segment*	0006E02^1^	8q14	9q	————-
*B3GAT2*	*beta-1,3 glucuronyltransferase2 (glucuronosyltransferaseS)*	60B09^1^	8q14	————-	6q12
*D9S16 (CSSM025)*	*DNA segment*	016H12^1^	8q16	9q17-q21	————-
*ASCC3*	*activating signal cointegrator1 complex subunit3*	914D12^1^	8q21.2	————-	6q16
*D9S55 (BMS345)*	*DNA segment*	0163E12^1^	8q22	9q	————-
*RARSL*	*arginyl-tRNA synthetase-like*	890B11^2^	8q24	————-	6q16.1
*CYP11B1*	*cytochrome P450, family 11, subfamily B, polypeptide 1*	115F11^2^	9q13	14q13	8q21-q22
*D14S19 (RM180)*	*DNA segment*	(0517E01)-517G01^1^	9q15	14q	————-
*D14S47 (BMS1941)*	*DNA segment*	(0234A01)239A01^2^	9q17	14q	————-
*BRCA2*	*breast cancer 2, early onset*	334F1^2^	10q15	12q15	13q12-q13
*ACACA*	*acetyl-Coenzyme A carboxylase alpha*	42D12^2^	11q13	19q13	17q21
*MYH2*	*myosin, heavy polypeptide 2, skeletal muscle, adult*	120C2^2^	11q17	19q15-q16	17p13.1
*LAMC2*	*laminin, gamma 2*	191B7^2^	12q23	16q23	1q25-q31
*ITGB1*	*integrin, beta 1 (fibronectin receptor, beta polypeptide, antigen CD29 includes MDF2, MSK12)*	132H1^2^	13q13dist	13q13dist	10p11.2
*RAB18*	*RAB18, member RAS oncogene family*	554C8^2^	13q15prox	13q15prox	10
*PSMA7*	*proteasome (prosome, macropain) subunit, alpha type, 7*	946B2^2^	13q22 prox	13q22 prox	20
*DPEP1*	*dipeptidase 1 (renal)*	262F4^2^	14q13	18q13	16q24
*MC1R*	*melanocortin 1 receptor (alpha melanocyte stimulating hormone receptor)*	132F3^2^	14q13	18q13	16q24.3
*GNAO1*	*guanine nucleotide binding protein (G protein), alpha activating activity polypeptide O*	527F2+657E3^2^	14q15	18q15	16
*PTGIR*	*prostaglandin I2 (prostacyclin) receptor (IP)*	276E4^2^	14q24dist	18q24dist	19q13.3
*SLC6A3*	*solute carrier family 6 (neurotransmitter transporter, dopamine), member 3*	72F4^2^	16q24	20q24	5p15.3
*IL2*	*interleukin 2*	129G5^2^	17q22	17q22dist	4q26-q27
*NOS1*	*nitric oxide synthase 1 (neuronal)*	208D8^2^	17q24	17q25	12q14-qter
*COMT*	*catechol-O-methyltransferase*	475C7^2^	17q26	17q26	22q11.21-q11.23
*MITF*	*microphthalmia-associated transcription factor*	52G5^2^	19q22	22q22	3p14.1-p12.3
*PBX2P1* (old alias: *PBXP1*)	*pre-B-cell leukaemia transcription factor pseudogene 1*	130G12^2^	19q22	22q22	3q23-q24
*BRD2*	*bromodomain containing2*	948D01^1^	20q13	————-	6p21.3
*POLR1C*	*polymerase(RNA) I polypeptide C, 30 Kda*	237C05^1^	20q15dist	————-	6p21.1
*PPP2R5D*	*protein phosphatase2, regulatory subunit B (B56),delta isoform*	364A09^1^	20q15dist	————-	6p21.1
*TNRC5*	*trinucleotide repeat containing 5*	364A09^1^	20q15dist	————-	6pter-p12.1
*BAT2*	*HLA-B associated transcript2*	660D10^1^	20q22prox	————-	6p21.3
*BAT4*	*HLA-B associated transcript4*	660D10^1^	20q22prox	————-	6p21.3
*C4B*	*complement component 4B*	573A10^1^	20q22	23q12d-q13p	6p21.3
*HSPA1B* (old alias:*HSP70-2*)	*heat shock 70 kD protein 2*	0573C02^1^	20q22	23q22	6p21.3
*CDC5L*	*CDC5 cell division cycle 5-like (*S. pombe*)*	192C02^1^	20q22	————-	6p
*OLA-DRA2*	*major histocompatibility complex, class II, DR alpha*	589B09^1^	20q22	————-	6p21.3
*D29S35 (BMS1112)*	*DNA Segment*	0133G06^1^	21q13	29	————-
*LDHA*	*lactate dehydrogenase A*	0039C07^1^	21q22	29q22	11p15.1
*DNTT*	*deoxynucleotidyltransferase, terminal*	169D3^2^	22q21	26q21	10q23-q24
*PAX2*	*paired box gene 2*	99A10^2^	22q21dist	26q21	10q25
*OAT*	*ornithine aminotransferase (gyrate atrophy)*	84B5^2^	22q23 dist	26q23prox	10q26
*CYB5A* (old alias: *CYB5*)	*cytochrome b5 type A (microsomal)*	369C2^2^	23q12	24q12	18q23
*DSG2*	*desmoglein 2*	312D1^2^	23q21	24q21-q22	18q12.1
*F11*	*coagulation factor XI (coagulation factor 11) (plasma thromboplastin antecedent)*	334A10^2^	26q15	27q15	4q35

Bovine

caprine

BAC clones, as well as comparisons with both cattle (BTA) (BovMap; Hayes *et al.* 2003) and human (HSA) (HUGO, known genes) chromosome locations are reported.

Labelling of probes was done with biotin or digoxigenin with BRL-Gibco and Roche kits respectively. Ethanol precipitation was carried out in the presence of bovine COT-1 DNA or caprine genomic DNA for bovine and caprine BAC clones respectively to suppress repetitive sequences. *In situ* hybridization, signal detection, chromosome staining, microscope observation and image processing were described before (Di Meo *et al.* 2005). At least 20 metaphases were examined for each probe. Chromosome identification and band nomenclature for sheep chromosomes followed the R-banded standard ideogram reported in the latest international chromosome nomenclature (ISCNDB 2001). Only loci assigned to specific chromosome bands or regions in the present and previous studies, as well as those reported in SheepBase and references therein), were considered. Symbols of type I and type II loci followed HUGO (http://www.gene.ucl.ac.uk/nomenclature/) and BovMap (http://locus.jouy.inra.fr/cgi-bin/bovmap/intro2.pl) nomenclatures respectively. GoatMap data were fromhttp://locus.jouy.inra.fr/cgi-bin/lgbc/mapping/common/main.pl?BASE = goat.

## Results and discussion

The frequency of hybridization signals on both chromosomes and chromatids, or on a single chromosome or chromatid, varied between 35% (*ASCC3*) and 81% (*UMPS*). All mapped loci were localized on homologous ovine chromosomes and chromosome bands when compared with cattle and goat positions. A few apparent differences between published and expected localizations were due to the banding techniques used in different studies. The data confirmed the high conservation of autosomal chromosomes among the bovid species. Loci FISH-mapped in the present study with locus name and symbol, clone identification and chromosome localization in sheep, cattle, goats and humans are listed in Table 1. Of these loci, 11 (*B3GAT2, ASCC3, RARSL, BRD2, POLR1C, PPP2R5D, TNRC5, BAT2, BAT4, CDC5L* and *OLA-DRA*) have been FISH-mapped in sheep only. An additional 11 loci (*D3S32, D1S86, BMS2621, SFXN5, D5S3, D5S68, CSKB1, D7S49, D9S15, D9S55* and *D29S35*) were assigned to specific sheep chromosome bands but only to whole chromosomes in cattle and goat (Table 1). Ten loci (*BRD2, POLR1C, PPP2R5D, TNRC5, BAT2, BAT4, C4B, HSPA1B, CDC5L* and *OLA-DRA*) were assigned to OAR20 extending the physical organization of this chromosome, which contains the major histocompatibility complex of sheep.

**Figure 1:**
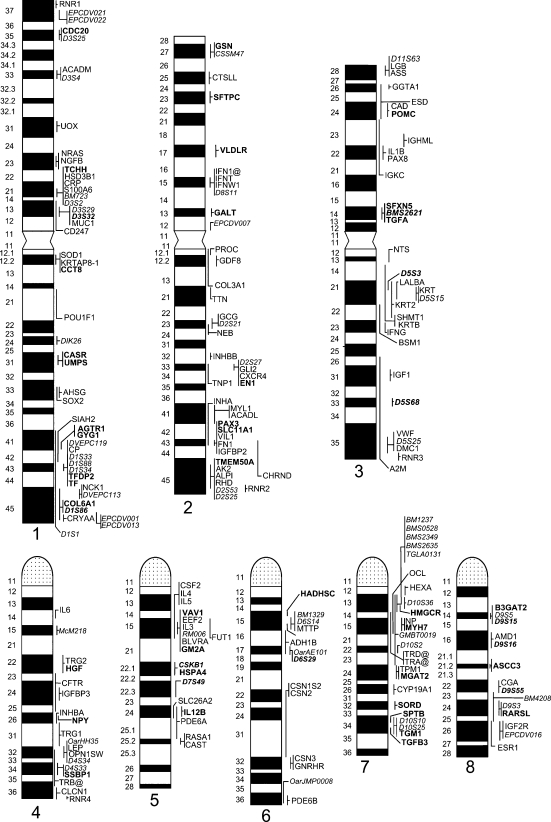

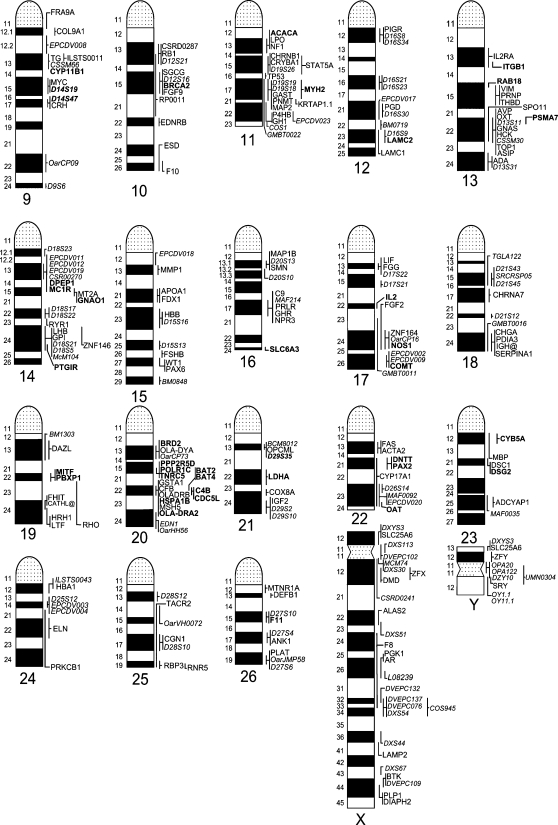
The new comprehensive sheep cytogenetic map on the latest standard R-banded ideograms (ISCNDB 2001). The 452 loci include 291 type I loci (presented in normal characters) and 161 type II loci (in italics). Also see. Loci mapped in the present study are reported in bold.

The revised sheep cytogenetic map, including loci previously mapped to specific chromosome bands or regions and the loci mapped in the present study on standard R-banded ideograms, is shown in Fig. 1. A total of 452 loci were assigned to specific chromosome bands or regions of sheep chromosomes, of which 291 are type I and 161 are type II, extending the cytogenetic map and density of markers available for this economically important species. These loci are also listed in, which includes the localization of all FISH-mapped loci in sheep and cattle and/or goat, the bovine syntenic groups and references.

Comparative mapping with human (Table 1) confirms previous comparative mapping data available from BovMap, SheepBase and GoatBase. Alignment of the cytogenetic map locations of loci between sheep, cattle and goats confirms the high degree of autosomal chromosome conservation among these bovid species, although some major discrepancies in the location of loci between OAR and BTA (or CHI) were observed: *TNP1* (OAR2q33-q34, BTA2q42-q43), *KRT1* (OAR3q21, CHI5q25), *HEXA* (OAR7q12, BTA10q15dist), *ANK1* (OAR26q17, CHI27q19) and *D9S6* (OAR9q24, CHI9q26). The assignment of *D9S6* merits further investigation because OAR9 is currently designated as homologous to CHI14, not CHI9 (ISCNDB 2001). Other minor discrepancies were noted (designated as more than two bands of difference) for *D6S29* (OAR6q17, BTA6q22) and *C4B* (OAR20q22, BTA23q12-q13).

Cattle, sheep and goat autosomes have been arranged using only one common chromosome banding system in the latest chromosome nomenclature of bovid species (ISCNDB 2001), as a result of their high chromosome banding similarities. In addition, all 31 bovine (and ovine/caprine) syntenic groups have been definitively assigned to specific chromosomes on the basis of official marker assignments performed with both G/Q and R-banded chromosomes of cattle (Hayes *et al.* 2000), as well as in those of both sheep and goat chromosomes (Di Meo *et al.* 2003). The use of a common chromosome banding system among chromosome of bovid species allows easier comparison of physical maps.

The new cytogenetic map presented here will be a useful tool for further studies on both molecular and clinical cytogenetics of this species. In addition it will allow a better anchoring of linkage (Maddox *et al.* 2001) and future RH maps by providing independent evidence for the localization and orientation of markers on specific chromosome regions. With few exceptions, all sheep chromosomal bands have at least one locus in the ovine cytogenetic map described here. Use of this cytogenetic mapping data, along with linkage and RH-mapping information, will greatly advance our understanding of the physical organization of the sheep genome and provide a sound platform for local and complete sequencing of the sheep genome.

## Supplementary Material

The following supplementary material is available for this article online from http://www.blackwell-synergy.com/doi/full/10.1111/j.1365-2052.2007.01598.x

Table S1 Loci assigned to chromosome bands or regions in sheep (OAR), compared to bovine (BTA) and goat (CHI) assignments.
