# Supplementary material for: An advanced sheep (Ovis aries, 2n = 54) cytogenetic map and assignment of 88 new autosomal loci by fluorescence in situ hybridization and R-banding
Source: Anim Genet. 2007 Jun 1;38(3):233–40. doi: 10.1111/j.1365-2052.2007.01598.x (PMC2063634; doi:10.1111/j.1365-2052.2007.01598.x)
Supplement: Table S1 — Loci assigned to chromosome bands or regions in sheep (OAR), compared to bovine (BTA) and goat (CHI) assignments. [file age0038-0233_TableS1.doc]

| **Supplemental Table 1.** Loci assigned to chromosome bands or regions in sheep (OAR), compared to bovine (BTA) (BovMap; Hayes *et al*. 2003) and goat (CHI) (GoatMap) assignments. Conventional locus symbols are reported between parenthesis. | | | | | | |
| --- | --- | --- | --- | --- | --- | --- |
| Locus symbol | Locus name | | OAR | BTA\CHI* | U^1^ | References |
| *CD247*  *(CD3Z)* | *CD247 molecule* | | 1p11-14 | 3q11-q14 | 6 | Ansari *et al.* 1994 |
| *MUC1* | *mucin 1, cell surface associated* | | 1p13 | 3q13 | 6 | Perucatti *et al.* 2006a |
| *D3S32*  *(ILSTS096)* | *DNA segment* | | 1p13 | 3 | 6 | present study |
| *D3S29*  *(IDVGA53)* | *DNA segment* | | 1p13 | 3q21 | 6 | Di Meo *et al.* 2002 |
| *TCHH*  *(THH)* | *trichohyalin* | | 1p21 | 3q21 | 6 | present study |
| *D3S2*  *(CSSM054)* | *DNA segment* | | 1p21 | 3 | 6 | Iannuzzi *et al.* 2003a |
| *BM723* | *DNA segment* | | 1p21 | -------------- | 6 | Iannuzzi *et al.* 2003a |
| *S100A6*  *(CACY)* | *S100 calcium-binding protein A6* | | 1p21 | 3q21* | 6 | Iannuzz *et al.* 2000b |
| *CRP* | *C-reactive protein, pentraxin-related* | | 1p21 | 3q13 | 6 | Iannuzzi *et al.* 2000b |
| *HSD3B1* | *Hydroxy-delta-5-steroid dehydrogenase, 3 beta- and steroid delta-isomerase 1* | | 1p21 | 3q21 | 6 | Di Meo *et al.*  2003 |
| *NGFB* | *Nerve growth factor, beta polypeptide* | | 1p23 | 3q23 | 6 | Iannuzzi *et al.* 2000b |
| *NRAS* | *neuroblastoma RAS viral (v-ras) oncogene homolog* | | 1p23 | 3 | 6 | Iannuzzi *et al.* 2000b |
| *UOX* | *Urate oxidase* | | 1p31 | 3q31-q32.1 | 6 | Iannuzzi *et al.* 2000b |
| *D3S4*  *(HUJ177)* | *DNA segment* | | 1p33 | 3 | 6 | Iannuzzi *et al.* 2003a |
| *ACADM* | *acyl-Coenzyme A dehydrogenase, C-4 to C-12 straight chain* | | 1p33 | 3 | 6 | Iannuzzi *et al.* 2000b |
| *D3S25*  *(IDVGA35)* | *DNA segment* | | 1p35 | 3q35 | 6 | Di Meo *et al.* 2002 |
| *EPCDV021* | *DNA segment* | | 1p37 | -------------- | 6 | SheepBase^2^ |
| *EPCDV022* | *DNA segment* | | 1p37 | -------------- | 6 | SheepBase |
| *RNR1* | *RNA, ribosomal 1* | | 1p37 | -------------- | 6 | SheepBase |
| *CDC20* | *CDC20 cell division cycle 20 homolog (S. cerevisiae)* | | 1p35 | 3q35 | 6 | present study |
| *CCT8* | *chaperonin containing TCP1, subunit 8 (theta)* | | 1q12.2 | 1q12.2 | 10 | present study |
| *SOD1* | *superoxide dismutase 1, soluble (amyotrophic lateral sclerosis 1 (adult)* | | 1q12.2 | 1q12.2 | 10 | Di Meo *et al.* 2003 |
| *KRTAP8-1* | *keratin associated protein 8-1* | | 1q12.2 | 1q12.2 | 10 | Iannuzzi *et al.* 2000b |
| *POU1F1* | *POU domain, class 1, transcription factor 1 (Pit1, grouth hormone factor 1)* | | 1q21-q22 | 1q21-q22 | 10 | SheepBase |
| *DIK26*  *(411G12)* | *DNA segment* | | 1q24 | 1 | 10 | Iannuzzi *et al.* 2003a |
| *UMPS* | *uridine monophosphate synthetase (orotate phosphoribosyl transferase and orotidine-5'-decarboxylase)* | | 1q31 | 1q31 | 10 | present study |
| *CASR* | *calcium-sensing receptor (hypocalciuric hypercalcemia 1, severe neonatal hyperparathyroidism)* | | 1q31 | 1q31 | 10 | present ptudy |
| *AHSG* | *alpha-2-HS-glycoprotein* | | 1q33 | 1q33-q34* | 10 | Iannuzzi *et al*. 2000b |
| *SOX2* | *SRY (sex determining region Y)-box 2* | | 1q33 | 1q33 | 10 | Hayes *et al.* 1996 |
| *SIAH2* | *seven in absentia homolog 2 (Drosophila)* | | 1q41-q42 | ----------- | 10 | [Mandon-Pepin](http://www.ncbi.nlm.nih.gov/entrez/query.fcgi?db=pubmed&cmd=Search&itool=pubmed_Abstract&term="Mandon-Pepin+B"%5BAuthor%5D) *et al.* 2003 |
| *GYG1*  *(GYG)* | *glycogenin 1* | | 1q41dist | 1q42 | 10 | present study |
| *AGTR1* | *angiotensin II receptor, type 1* | | 1q41dist. | 1q42 | 10 | present study |
| *D1S1*  *(CSRD1613)* | *DNA segment* | | 1q41-q45 | 1q36-qter | 10 | SheepBase |
| *DVEPC119* | *DNA segment* | | 1q43 | 1q44-q45 | 10 | Iannuzzi *et al.* 2003a |
| *CP* | *ceruloplasmin ( ferroxidase)* | | 1q43 | 1q41dist | 10 | Iannuzzi *et al.* 2000b |
| *D1S88*  *(BMS1757)* | *DNA segment* | | 1q43 | 1 | 10 | Iannuzzi *et al.* 2003a |
| *D1S34*  *(BM1824)* | *DNA segment* | | 1q43 | 1 | 10 | SheepBase |
| *D1S33*  *(BL28)* | *DNA segment* | | 1q43 | 1q42 | 10 | Iannuzzi *et al.* 2003a |
| *TFDP2* | *transcription factor Dp-2 (E2F dimerization partner 2)* | | 1q43 | 1q43prox | 10 | present study |
| *TF* | *transferrin* | | 1q43dist | 1q43dist | 10 | present study |
| *NCK1* | *NCK adaptor protein 1* | | 1q45 | 1 | 10 | Iannuzzi *et al.* 2000b |
| *DVEPC113* | *DNA segment* | | 1q45 | 1 | 10 | Iannuzzi *et al.* 2003a |
| *COL6A1* | *collagen, type VI, alpha 1* | | 1q45 | 1q45 | 10 | present study |
| *D1S86*  *(BMS922)* | *DNA segment* | | 1q45 | ------------- | 10 | present study |
| *CRYAA* | *crystallin alpha A* | | 1q45 | 1q45* | 10 | Iannuzzi *et al.* 2000b |
| *EPCDV001* | *DNA segment* | | 1q45 | --------------- | 10 | SheepBase |
| *EPCDV013* | *DNA segment* | | 1q45 | --------------- | 10 | SheepBase |
| *EPCDV007* | *DNA segment* | | 2p12 | ---------------- | 18 | SheepBase |
| *GALT* | *Galactose-1-phosphate uridylyltransferase* | | 2p13 | 8q13 | 18 | present study |
| *D8S11*  *(TGLA010)* | *DNA segment* | | 2p15 | 8 | 18 | Iannuzzi *et al.* 2003a |
| *IFNW1* | *Interferon, omega 1* | | 2p15 | 8q15 | 18 | Iannuzzi *et al.* 1993 |
| *IFNT* | *trophoblast interferon* | | 2p15 | 8q15 | 18 | Iannuzzi *et al.* 1993 |
| *IFN1@* | *interferon, type 1, cluster* | | 2p15 | 8q15 | 18 | Di Meo *et al.* 2003 |
| *VLDLR* | *very low density lipoprotein receptor* | | 2p17 | 8q17 | 18 | present study |
| *SFTPC* | *surfactant, pulmonary-associated protein C* | | 2p23prox | 8q21dist | 18 | present Study |
| *CTSLL1* | *cathepsin L-like 1* | | 2p25 | 8q25-q26* | 18 | Iannuzzi *et al.* 2001b |
| *CSSM47* | *DNA segment* | | 2p27 | 8q27* | 18 | Iannuzzi *et al.* 2003a |
| *GSN* | *gelsolin (amyloidosis, Finnish type)* | | 2p27dist | 8q28 | 18 | present study |
| *PROC* | *protein C (inactivator of coagulation factors Va and VIIIa)* | | 2q12.1 | 2q12 | 17 | Lopez-Corrales *et al.* 1998 |
| *GDF8* | *growth differentiation factor 8* | | 2q12.2 | 2 | 17 | Di Meo *et al.* 2006 |
| *COL3A1* | *collagen, type III, alpha 1 (Ehlers-Danlos syndrome type IV, autosomal dominant)* | | 2q12-13 | 2q12 | 17 | SheepBase |
| *TTN* | *titin* | | 2q21prox | 2q21 | 17 | Di Meo *et al.* 2006 |
| *GCG* | *glucagon* | | 2q23 | 2 | 17 | Di Meo *et al.* 2006 |
| *D2S21*  *(OarFCB020)* | *DNA segment* | | 2q23 | 2 | 17 | Iannuzzi *et al.* 2003a |
| *NEB* | *nebulin* | | 2q23-24 | 2 | 17 | Di Meo *et al.* 2006 |
| *CXCR4*  *(NPY3R )* | *chemokine (C-X-C motif), receptor 4* | | 2q33 | 2 | 17 | Di Meo *et al.* 2006 |
| *MYL1* | *myosin, light polypeptide 1, alkali; skeletal, fast* | | 2q41 | 2q41prox | 17 | Di Meo *et al.* 2006 |
| *ACADL* | *acyl-Coenzyme A dehydrogenase, long chain* | | 2q41prox | 2q41 | 17 | Di Meo *et al.* 2006 |
| *INHBB* | *inhibin, beta B (activin AB beta polypeptide)* | | 2q31-33 | 2q31-q33 | 17 | SheepBase |
| *D2S27*  *(ILSTS082)* | *DNA segment* | | 2q33 | 2 | 17 | Iannuzzi *et al.* 2003a |
| *GLI2* | *gli-kruppel family member GLI2* | | 2q33 | 2q33 | 17 | Iannuzzi *et al.* 2001b |
| *EN1* | *Engrailed homolog 1* | | 2q33 | 2q33 | 17 | present study |
| *TNP1* | *transition protein 1 (during histione to protamine replacement)* | | 2q33-q34 | 2q42-q43 | 17 | Lopez-Corrales *et al.* 1998 |
| *INHA* | *inhibin, alpha* | | 2q41-q43 | 2q36-q42 | 17 | SheepBase |
| *SLC11A1*  *(NRAMP1)* | *solute carrier family 11 (proton-coupled divalent metal ion transporters), member 1* | | 2q43 | 2q43 | 17 | present study |
| *VIL1* | *villin 1* | | 2q43 | 2q43 | 17 | Di Meo *et al.* 2003 |
| *IGFBP2* | *Insuline-Like grow factor binding protein,2 36kDa* | | 2q43 | 2 | 17 | Di Meo *et al.* 2006 |
| *PAX3* | *Paired box gene 3 (waardenburg syndrome)* | | 2q43 | 2q43 | 17 | present study |
| *FN1* | *Fibronectin1* | | 2q43 | 2 | 17 | Di Meo *et al.* 2006 |
| *AK2* | *adenylate kinase 2* | | 2q45 | 2 | 17 | Iannuzzi *et al.* 2001b |
| *ALPI* | *alkaline phosphatase, intestinal* | | 2q45 | 2q45 | 17 | Lopez-Corrales *et al.* 1998 |
| *CHRND* | *cholinergic receptor, nicotinic delta* | | 2q43-q45 | ------- | 17 | SheepBase |
| *RHD* | *Rh blood groups, D antigen* | | 2q45 | 2q45 | 17 | Iannuzzi *et al.* 2001b |
| *D2S53*  *(INRA231)* | *DNA segment* | | 2q45 | 2 | 17 | Iannuzzi *et al.* 2003a |
| *D2S25*  *(IDVGA64)* | *DNA segment* | | 2q45 | 2q44 | 17 | Iannuzzi *et al.* 2003a |
| *TMEM50A*  *(SMP1)* | *transmembrane protein 50A* | | 2q45 | 2q45prox | 17 | present study |
| *RNR2* | *RNA, ribosomal 2* | | 2q45 | -------------- | 17 | SheepBase |
| *BMS2621* | *DNA segment* | | 3p14 | 11 | 16 | present study |
| *SFXN5* | *Sideroflexin 5* | | 3p14 | ------------ | 16 | present study |
| *TGFA* | *transforming growth factor, alpha* | | 3p14 | 11q14 | 16 | present study |
| *IGKC* | *immunoglobulin kappa constant* | | 3p21-q23 | -------------- | 16 | SheepBase |
| *IL1B* | *interleukin 1, beta* | | 3p22 | 11q22-q24 | 16 | Lopez-Corrales *et al.* 1998 |
| *PAX8* | *paired box gene 8* | | 3p22^3^ | 11q22**^3^** | 16 | SheepBase |
| *IGHML* | *Immunoglobulin heavy chain M-like* | | 3p23 | 11q23 | 16 | SheepBase |
| *CAD* | *carbamoyl-phosphate synthetase 2, aspartate transcarbamylase, and dihydroorotase* | | 3p24 | 11q24* | 16 | Iannuzzi *et al.* 2001b |
| *POMC* | *proopiomelanocortin (adrenocorticotropin/ beta-lipotropin/ alpha-melanocyte stimulating hormone/ beta-melanocyte stimulating hormone/ beta-endorphin)* | | 3p24 | 11q24dist | 16 | present study |
| *ESD* | *Esterase D/formylglutathione hydrolase* | | 3p24-p26 | ------------- | 16 | Graphodatsky *et al.* 1993 |
| *GGTA1* | *glycoprotein, alpha-galactosyltransferase 1* | | 3p26 | 11q26 | 16 | Hayes *et al.* 1996 |
| *ASS1* | *Arginosuccinate synthetase 1* | | 3p28 | 11q28prox | 16 | Iannuzzi *et al.* 2001b |
| *D11S63*  *(ILSTS028)* | *DNA segment* | | 3p28 | 11 | 16 | Iannuzzi *et al.* 2003a |
| *LGB* | *lactoglobulin, beta* | | 3p28 | 11q28 | 16 | Di Meo *et al.* 2003 |
| *NTS* | *neurotensin* | | 3q12-14 | 5q13 | 3 | Wood *et al.* 1993 |
| *KRTB* | *Dna segment* | | 3q14-22 | 5q14-q22 | 3 | Hediger *et al.* 1991 |
| *D5S3*  *(ETH10)* | *Dna segment* | | 3q21prox | 5 | 3 | present study |
| *LALBA* | *lactalbumin, alpha-* | | 3q21 | 5q21 | 3 | SheepBase |
| *KRT1* | *keratin 1 (epidermolytic hyperkeratosis)* | | 3q21 | 5q25* | 3 | Di Meo *et al.* 2000 |
| *D5S15*  *(BMC1009)* | *DNA segment* | | 3q21 | 5q23 | 3 | Iannuzzi *et al.* 2003a |
| *KRT2* | *keratin 2 (epidermal ichthyosis bullosa of Siemens)* | | 3q21-q22 | -------------- | 3 | SheepBase |
| *SHMT1* | *serine hydroxymethyltransferase 1 (soluble)* | | 3q14-q22 | -------------- | 3 | SheepBase |
| *BSM1* | *Bovine submaxillary mucin gene* | | 3q2.2-q2.3 | 5q2.2-q2.3 | 3 | Vozdova *et al.* 2003 |
| *IFNG* | *interferon gamma* | | 3q23 | 5q23 | 3 | Di Meo *et al.* 2003 |
| *A2M* | *alpha-2-macroglobulin* | | 3q26-q35 | 5 | 3 | Graphodatsky *et al.* 1993 |
| *IGF1* | *insulin-like growth factor 1 (somatomedin C)* | | 3q31 | 5q31* | 3 | Di Meo *et al.* 2000 |
| *D5S68*  *(BMS1658)* | *DNA segment* | | 3q33 | -------------- | 3 | present study |
| *DMC1* | *DMC1 dosage suppressor of mck1 homolog, meiosis-specific homologous recombination (yeast)* | | 3q35 | ----------------- | 3 | [Mandon-Pepin](http://www.ncbi.nlm.nih.gov/entrez/query.fcgi?db=pubmed&cmd=Search&itool=pubmed_Abstract&term="Mandon-Pepin+B"%5BAuthor%5D) *et al.* 2003 |
| *VWF* | *von Willebrand factor* | | 3q35 | 5q35 | 3 | SheepBase |
| *D5S25*  *(BM2830)* | *DNA segment* | | 3q35 | 5 | 3 | Iannuzzi *et al.* 2003a |
| *RNR3* | *RNA, ribosomal 3* | | 3q35 | --------------- | 3 | SheepBase |
| *IL6* | *interleukin 6 (interferon, beta 2)* | | 4q14 | 4 | 13 | SheepBase |
| *McM218* | *DNA segment* | | 4q15 | -------------- | 13 | Iannuzzi *et al.* 2003a |
| *TRG2* | *tRNA glycine 2* | | 4q22 | -------------- | 13 | Antonacci *et al.* 2006 |
| *HGF* | *hepatocyte growth factor (hepapoietin A; scatter factor)* | | 4q22 | 4q15dist-21 | 13 | present study |
| *TRG1* | *tRNA glycine (CCC) 1* | | 4q31 | ------------- | 13 | Antonacci *et al.* 2006 |
| *CFTR* | *cystic fibrosis transmembrane conductance regulator ( ATP-binding cassette sub-family C, member 7)* | | 4q23-q25 | 4q23-q25 | 13 | SheepBase |
| *IGFBP3* | *Insulin-like growth factor binding protein-3* | | 4q26 | 4q26* | 13 | Di Meo *et al.* 2000 |
| *NPY* | *neuropeptide Y* | | 4q26prox | 4q25-26 | 13 | present study |
| *INHBA* | *inhibin, beta A (activin A, activin AB alpha polypeptide)* | | 4q26 | 4q26 | 13 | Di Meo *et al.* 2003 |
| *OarHH35* | *DNA segment* | | 4q32 | 4q31* | 13 | Iannuzzi *et al.* 2003a |
| *LEP* | *leptin (obesity homolog, mouse)* | | 4q32 | 4q32 | 13 | Perucatti *et al.* 2006b |
| *OPN1SW*  *(BCP)* | *opsin 1 (cone pigments), short-wave-sensitive (color blindness, tritan)* | | 4q32 | 4q32* | 13 | Di Meo *et al.* 2000 |
| *D4S34*  *(IDVGA84)* | *DNA segment* | | 4q32 | 4q32 | 13 | Di Meo *et al.* 2002 |
| *SSBP1* | *single-stranded DNA binding protein 1* | | 4q34dist. | 4q34dist | 13 | present study |
| *D4S33*  *(IDVGA61)* | *DNA segment* | | 4q34 | 4q32 | 13 | Di Meo *et al.* 2002 |
| *TRB@*  *(TCRB)* | *T cell receptor beta locus* | | 4q32-qter | ------------- | 13 | Pearce *et al.* 1995 |
| *CLCN1* | *chloride channel 1, skeletal muscle (Thomsen disease, autosomal dominant)* | | 4q34 | 4 | 13 | Di Meo *et al.* 2000 |
| *RNR4* | *RNA, ribosomal 4* | | 4q36 | -------------- | 13 | SheepBase |
| *CSF2* | *colony stimulating factor 2 (granulocyte-macrophage)* | | 5q13-q15 | 7 | 22 | Sheep Base |
| *IL4* | *interleukin 4* | | 5q13-q15 | 7q15-q21 | 22 | Sheep Base |
| *IL5* | *interleukin 5 (colony-stimulating factor, eosinophil)* | | 5q13-q15 | 7q15-q21 | 22 | Sheep Base |
| *BLVRA*  *(BLVR)* | *biliverdin reductase A* | | 5q15 | 7q15 | 22 | Popescu *et al.* 1995 |
| *VAV1* | *vav 1 oncogene* | | 5q15prox | 7q15prox | 22 | present study |
| *EEF2* | *eukaryotic translation elongation factor 2* | | 5q15 | 7q15 | 22 | Hayes *et al.* 1996 |
| *IL3* | *interleukin 3 (colony-stimulating factor, multiple)* | | 5q15 | 7q15-q21 | 22 | Iannuzzi *et al.* 2003a |
| *RM006* | *DNA segment* | | 5q15 | 7q15* | 22 | Iannuzzi *et al.* 2003a |
| *FUT1* | *fucosyltransferase 1(galactoside 2- alpha-L-fucosyltranferase, H blood group)* | | 5q15 | 7q15 | 22 | SheepBase |
| *GM2A* | *GM2 ganglioside activator* | | 5q15 | 7q21 | 22 | present study |
| *CSKB1* | *DNA segment* | | 5q22.1 | 7 | 22 | present study |
| *HSPA4* | *heat shock 70kDa protein 4* | | 5q22.1 | 7q22.1 | 22 | present study |
| *D7S49*  *(BMS792)*  *(D0S246)* | *DNA segment* | | 5q22.3 | 7 | 22 | present study |
| *SLC26A2*  *(DTDST)* | *solute carrier family 26 (sulfate transporter), member 2* | | 5q24 | 7q24 | 22 | Perucatti *et al.* 2006b |
| *IL12B* | *interleukin 12B [natural killer cell stimulatory factor 2, cytotoxic lymphocyte maturation factor 2, p40]* | | 5q24 | 7q23-q24 | 22 | present study |
| *PDE6A*  *(PDEA)* | *phosphodiesterase 6A, cGMP-specific, rod, alpha* | | 5q23-q31 | -------------- | 22 | Pearce *et al.* 1995 |
| *RASA1* | *RAS p21 protein activator (GTPase activating protein) 1* | | 5q25.2 | 7q25.2 | 22 | Di Meo *et al.* 2003 |
| *CAST* | *calpastatin* | | 5q25.2 | 7q27 | 22 | Iannuzzi *et al.* 2001b |
| *HADH*  *(HADHSC)* | *hydroxyacyl-Coenzyme A dehydrogenase* | | 6q15prox | 6q15prox | 15 | present study |
| *BM1329* | *DNA segment* | | 6q15 | 6q15* | 15 | SheepBase |
| *D6S14*  *(BM1329)* | *DNA segment* | | 6q15 |  | 15 | Iannuzzi *et al.* 2003a |
| *MTTP*  *(MTP)* | *microsomal triglyceride transfer protein* | | 6q15 | 6q15* | 15 | Di Meo *et al.* 2000 |
| *D6S29*  *(IDVGA65)* | *DNA segment* | | 6q17 | 6q22 | 15 | present study |
| *ADH1B*  *(ADH2)* | *alcohol dehydrogenase IB (class I), beta polypeptide* | | 6q16-q17 | -------------- | 15 | SheepBase |
| *OarAE101* | *DNA segment* | | 6q17 | 6q18* | 15 | Iannuzzi *et al.* 2003a |
| *CSN1S2* | *alfa-S2 casein* | | 6q22-q31 | 6q31 | 15 | SheepBase |
| *CSN2* | *casein beta* | | 6q22-q31 | 6q32 | 15 | Broad *et al.* 1994 |
| *CSN3*  *(CSN10)* | *casein kappa* | | 6q32 | 6q32 | 15 | Di Meo *et al.* 2003 |
| *GNRHR* | *gonadotropin-releasing hormone receptor* | | 6q32 | 6q31* | 15 | Di Meo *et al.* 2000 |
| *OarJMP0008* | *DNA segment* | | 6q34-q36 | --------------- | 15 | SheepBase |
| *PDE6B* | *phosphodiesterase 6B, cGMP-specific, rod, beta (congenital stationary night blindness 3, autosomal dominant)* | | 6q36 | 6q36* | 15 | Di Meo *et al.* 2000 |
| *HEXA* | *hexosaminidase A (alpha polypeptide)* | | 7q12 | 10q15dist | 5 | SheepBase |
| *HMGCR* | *3-hydroxy-3-methylglutaryl-Coenzyme A reductase* | | 7q13prox | 10q12 | 5 | present study |
| *D10S36*  *(JAB10)* | *DNA segment* | | 7q13 | 10q15 | 5 | Di Meo *et al.* 2002 |
| *BM1237* | *DNA segment* | | 7q13 | -------------- | 5 | SheepBase |
| *BMS0528* | *DNA segment* | | 7q13 | -------------- | 5 | SheepBase |
| *BMS2349* | *DNA segment* | | 7q13 | -------------- | 5 | SheepBase |
| *BMS2635* | *DNA segment* | | 7q13 | -------------- | 5 | SheepBase |
| *TGLA0131* | *DNA segment* | | 7q13 | -------------- | 5 | SheepBase |
| *OCL* | *Ovine ceroid lipofuscinoses* | | 7q13-q15 | ------------- | 5 | Broom and Zhou, 2001 |
| *TRD@*  *(TCRD)* | *T cell receptor delta locus* | | 7q14-q22 | 10 | 5 | Massari *et al.* 1997 |
| *TRA@*  *(TCRA)* | *T cell receptor alpha locus* | | 7q14-q22 | 10 | 5 | Massari *et al.* 1997 |
| *MYH7* | *myosin, heavy chain 7, cardiac muscle, beta* | | 7q15 | 10q15-q21 | 5 | present study |
| *NP* | *nucleoside phosphorylase* | | 7q15 | 10q21* | 5 | Iannuzzi *et al.* 2001b |
| *GMBT0019* | *DNA segment* | | 7q13-q23 | -------------- | 5 | SheepBase |
| *D10S2*  *(TGLA378)* | *DNA segment* | | 7q22 | 10 | 5 | Iannuzzi *et al.* 2003a |
| *TPM1* | *tropomyosin 1 (alpha)* | | 7q24 | 10q26* | 5 | Iannuzzi *et al.* 2001b |
| *MGAT2* | *mannosyl (alpha-1,6-)-glycoprotein beta-1,2-N-acetylglucosaminyltransferase* | | 7q24 | 10q24 | 5 | present study |
| *CYP19A1*  *(CYP19)* | *cytochrome P450, family 19, subfamily A, polypeptide 1* | | 7q26 | 10q26 | 5 | Di Meo *et al.* 2003 |
| *SORD* | *sorbitol dehydrogenase* | | 7q32 | 10q32 | 5 | present study |
| *D10S25*  *(ILSTS005)* | *DNA segment* | | 7q34 | 10 | 5 | Iannuzzi *et al.* 2003a |
| *D10S10*  *(TGLA272)* | *DNA segment* | | 7q34 | 10 | 5 | Iannuzzi *et al.* 2003a |
| *TGM1* | *transglutaminase 1 (K polypeptide epidermal type I, protein-glutamine-gamma-glutamyltransferase)* | | 7q34 | 10q34 | 5 | present study |
| *SPTB* | *spectrin, beta, erythrocytic (includes spherocytosis, clinical type I)* | | 7q34prox | 10q34prox | 5 | present study |
| *TGFB3* | *transforming growth factor, beta 3* | | 7q34dist | 10q34dist | 5 | present study |
| *B3GAT2* | *beta-1,3 glucuronyltransferase2 (glucuronosyltransferaseS)* | | 8q14 | -------------- | 2 | present study |
| *D9S5*  *(INRA127)* | *DNA segment* | | 8q14 | 9 | 2 | Iannuzzi *et al.* 2003a |
| *D9S15*  *(BM2504)* | *DNA segment* | | 8q14 | 9 | 2 | present study |
| *AMD1* | *adenosylmethionine decarboxylase1* | | 8q16 | 9q16* ^3^ | 2 | Iannuzzi *et al.* 2001b |
| *D9S16*  *(CSSM025)* | *DNA segment* | | 8q16 | 9q17-q21 | 2 | present study |
| *ASCC3* | *Activating signal cointegrator1 complex subunit3* | | 8q21.2 | -------------- | 2 | present Study |
| *CGA* | *glicoprotein hormones, alpha polypeptide* | | 8q22 | 9 | 2 | Iannuzzi *et al.* 2001b |
| *D9S55*  *(BMS345)* | *DNA segment* | | 8q22 | 9 | 2 | present study |
| *BM4208* | *DNA segment* | | 8q23-q25 | --------------- | 2 | SheepBase |
| *D9S3*  *(TGLA73)* | *DNA segment* | | 8q24 | 9 | 2 | Iannuzzi *et al.* 2003a |
| *RARSL* | *Arginyl-tRNA synthetase-like* | | 8q24 | -------------- | 2 | present study |
| *IGF2R* | *insulin-like growth factor 2 receptor* | | 8q26 | 9q26 | 2 | Di Meo *et al.* 2003 |
| *EPCDV016* | *DNA segment* | | 8q26 | -------------- | 2 | SheepBase |
| *ESR1*  *(ESR)* | *estrogen receptor 1* | | 8q25-27 | 9 | 2 | Ansari *et al.* 1994 |
| *FRA9A* | *fragile site, folic acid type, rare, fra(9)(p21)* | | 9q11 | -------------- | 24 | SheepBase |
| *COL9A1* | *collagen type IX, alpha 1* | | 9q12.1 | 14q12* | 24 | Iannuzzi *et al.* 2001b |
| *EPCDV008* | *DNA segment* | | 9q12.2 | -------------- | 24 | SheepBase |
| *ILSTS0011* | *DNA segment* | | 9q12.2-13 | 14* | 24 | SheepBase |
| *TG* | *thyroglobulin* | | 9q13 | 14q13 | 24 | Di Meo *et al.* 2003 |
| *CYP11B1* | *cytochrome P450, family 11, subfamily B, polypeptide 1* | | 9q13 | 14q13 | 24 | present study |
| *CSSM66* | *DNA segment* | | 9q13 | 14q13* ^3^ | 24 | Iannuzzi *et al.* 2003a |
| *MYC* | *v-myc myelocytomatosis viral oncogene homolog (avian)* | | 9q15 | 14q16* | 24 | Iannuzzi *et al.* 2001b |
| *D14S19*  *(RM180)* | *DNA segment* | | 9q15 | 14 | 24 | present study |
| *D14S47*  *(BMS1941)* | *DNA segment* | | 9q17 | 14 | 24 | present study |
| *CRH* | *corticotropin releasing hormone* | | 9q17 | 14q19 | 24 | Iannuzzi *et al.* 2001b |
| *OarCP09* | *DNA segment* | | 9q22 | 14q24* | 24 | Iannuzzi et a., 2003a |
| *D9S6*  *(BM4208)* | *DNA segment* | | 9q24 | 9q26* | 24 | Iannuzzi *et al.* 2003a |
| *CSRD0287* | *DNA segment* | | 10q13 | -------------- | 27 | SheepBase |
| *RB1* | *retinoblastoma 1 (including osteosarcoma)* | | 10q13 | 12q13 | 27 | Di Meo *et al*. 2003 |
| *D12S21*  *(IDVGA57)* | *DNA segment* | | 10q13 | 12q13 | 27 | Di Meo *et al.* 2002 |
| *SGCG*  *(DAGA4)* | *sarcoglycan, gamma (35kDa dystrophin-associated glycoprotein*) | | 10q15 | 12q15-q21* | 27 | Iannuzzi *et al.* 2001b |
| *D12S16*  *(IDVGA41)* | *DNA segment* | | 10q15 | 12q15 | 27 | Di Meo *et al.* 2002 |
| *BRCA2* | *breast cancer 2, early onset* | | 10q15 | 12q15 | 27 | present study |
| *FGF9* | *fibroblast growth factor 9 (glia-activating factor)* | | 10q15 | ---------------- | 27 | [Mandon-Pepin](http://www.ncbi.nlm.nih.gov/entrez/query.fcgi?db=pubmed&cmd=Search&itool=pubmed_Abstract&term="Mandon-Pepin+B"%5BAuthor%5D) *et al.* 2003 |
| *RP0011*  *(RP11)* | *Ovine DNA segment, RP11* | | 10q13-q21 | -------------- | 27 | Sheep Base |
| *EDNRB* | *endothelin receptor type B* | | 10q22 | 12q22 | 27 | Iannuzzi *et al.* 2001b |
| *F10* | *coagulation factor X* | | 10q23-qter | 12 | 27 | Pearce *et al.* 1995 |
| *ESD* | *esterase D/formylglutathione hydrolase* | | 10q24-q26 | 12 | 27 | SheepBase |
| *LPO* | *lactoperoxydase* | | 11q13 | 19q13 | 21 | Iannuzzi *et al.* 2001b |
| *NF1* | *neurofibromin 1 (neurofibromatosis, von Recklinghausen disease, Watson disease)* | | 11q13 | 19q14 | 21 | Iannuzzi *et al.* 1999 |
| *ACACA* | *acetyl-Coenzyme A carboxylase alpha* | | 11q13 | 19q13 | 21 | present study |
| *CHRNB1* | *cholinergic receptor, nicotinic, beta polypeptide 1 (muscle)* | | 11q15 | 19q15 | 21 | Iannuzzi *et al.* 1999 |
| *D19S26*  *(IDVGA58)* | *DNA segment* | | 11q15 | 19q15 | 21 | Di Meo *et al.* 2002 |
| *STAT5A* | *signal transducer and activator of transcription 5A* | | 11q15 | 19q17 | 21 | SheepBase |
| *CRYBA1*  *(CRYB1)* | *crystallin, beta A1* | | 11q15 | 19q15 | 21 | Iannuzzi *et al*. 1999 |
| *TP53* | *tumor protein p53 (Li-Fraumeni syndrome)* | | 11q15-q16 | 19q15-q16 | 21 | Iannuzzi *et al.* 1999 |
| *D19S19*  *(IDVGA47)* | *DNA segment* | | 11q17 | 19q17 | 21 | Di Meo *et al.* 2002 |
| *D19S18*  *(IDVGA46)* | *DNA segment* | | 11q17 | 19q16 | 21 | Di Meo *et al.* 2002 |
| *GAST*  *(GAS)* | *gastrin* | | 11q17 | 19q17 | 21 | Iannuzzi *et al.*  2001b |
| *PNMT* | *phenylethanolamine N methyl transferase* | | 11q17 | 19q17* | 21 | Iannuzzi *et al.* 2001b |
| *MAP2* | *microtubule associated protein 2C* | | 11q17 | 19q21-q22* | 21 | Iannuzzi *et al.* 2001b |
| *MYH2* | *myosin, heavy chain 2, skeletal muscle, adult* | | 11q17 | 19q15-16 | 21 | present study |
| *KRTAP1-1* | *keratin associated protein 1-1* | | 11q21 | -------------- | 21 | SheepBase |
| *P4HB* | *procollagen-proline, 2-oxoglutarate 4-dioxygenase (proline 4-hydroxylase), beta polypeptide* | | 11q22 | 19q22 | 21 | Iannuzzi *et al.* 1999 |
| *GH1* | *growth hormone 1* | | 11q22 | 19q22 | 21 | Di Meo *et al.* 2003 |
| *EPCDV023* | *DNA segment* | | 11q22 | -------------- | 21 | SheepBase |
| *COS1* | *DNA segment* | | 11q23 | -------------- | 21 | SheepBase |
| *GMBT0022* | *DNA segment* | | 11q17-q23 | -------------- | 21 | SheepBase |
| *PIGR* | *polymeric immunoglobulin receptor* | | 12q12 | 16q12 | 1 | Di Meo *et al.* 2003 |
| *D16S8*  *(HUJ614)* | *DNA segment* | | 12q12 | 16 | 1 | Iannuzzi *et al.* 2003a |
| *D16S34*  *(IDVGA66)* | *DNA segment* | | 12q12 | 16q12 | 1 | Di Meo *et al.* 2002 |
| *D16S21*  *(IDVGA49)* | *DNA segment* | | 12q16 | 16q17 | 1 | Di Meo *et al.* 2002 |
| *D16S23*  *(IDVGA68)* | *DNA segment* | | 12q16 | 16q16 | 1 | Di Meo *et al.* 2002 |
| *EPCDV017* | *DNA segment* | | 12q21 | -------------- | 1 | SheepBase |
| *PGD* | *phosphogluconate dehydrogenase* | | 12q21 | 16q21* | 1 | Iannuzzi *et al.* 2001b |
| *D16S30*  *(IDVGA26)* | *DNA segment* | | 12q21 | 16q21 | 1 | Iannuzzi *et al.* 2003a |
| *BM0719* | *DNA segment* | | 12q22 | 16* | 1 | SheepBase |
| *D16S9*  *(HUJ625)* | *DNA segment* | | 12q23 | 16 | 1 | Iannuzzi *et al.* 2003a |
| *LAMC2* | *laminin, gamma 2* | | 12q23 | 16q23 | 1 | present study |
| *LAMC1* | *laminin, gamma 1 (formerly LAMB2)* | | 12q22-q24 | 16q22-q24 | 1 | Pearce *et al.* 1995 |
| *IL2RA* | *Interleukine 2receptor alfa* | | 13q13 | 13q13 | 11 | Di Meo *et al.* 2003 |
| *ITGB1* | *integrin, beta 1 (fibronectin receptor, beta polypeptide, antigen CD29 includes MDF2, MSK12)* | | 13q13dist | 13q13dist | 11 | present study |
| *RAB18* | *RAB18, member RAS oncogene family* | | 13q15prox | 13q15prox | 11 | present study |
| *VIM* | *vimentin* | | 13q15 | 13q16-q17 | 11 | Iannuzzi *et al.* 2001a |
| *PRNP* | *prion protein (p27-30) (Creutzfeld-Jakob disease, Gerstmann-Strausler-Scheinker syndrome, fatal familial insomnia)* | | 13q15 | 13q15 | 11 | Iannuzzi *et al.* 1998  Castiglioni *et al.* 1998 |
| *THBD* | *thrombomodulin* | | 13q15 | 13q17 | 11 | Iannuzzi *et al.* 2001a |
| *SPO11* | *SPO11 meiotic protein covalently bound to DSB homolog (S. cerevisiae)* | | 13q21-q22 | ----------- | 11 | [Mandon-Pepin](http://www.ncbi.nlm.nih.gov/entrez/query.fcgi?db=pubmed&cmd=Search&itool=pubmed_Abstract&term="Mandon-Pepin+B"%5BAuthor%5D) *et al.* 2003 |
| *AVP*  *(BIO154)* | *arginine vasopressin (neurophysin II, antidiuretic hormone, diabetes insipidus, neurohypophyseal)* | | 13q22 | 13q21-q22 | 11 | Iannuzzi *et al.* 2001a |
| *OXT* | *oxytocin, prepro- (neurophysin I)* | | 13q22 | 13q21-q22 | 11 | Iannuzzi *et al.* 2001a |
| *D13S11*  *(BL42)* | *DNA segment* | | 13q22 | 13q22 | 11 | Iannuzzi *et al.* 2001a |
| *GNAS*  *(GNAS1)* | *GNAS complex locus* | | 13q22 | 13q22 | 11 | Iannuzzi *et al.* 2001a |
| *HCK* | *haemopoietic cell kinase* | | 13q22 | 13q22 | 11 | Iannuzzi *et al.* 2001a |
| *CSSM30* | *DNA segment* | | 13q22 | 13q22* | 11 | Iannuzzi *et al.* 2001a |
| *TOP1* | *topoisomerase (DNA) I* | | 13q22 | 13q22-q23 | 11 | Iannuzzi *et al.* 2001a |
| *ASIP* | *agouti signaling protein, nonagouti homolog (mouse)* | | 13q22 | 13q22* | 11 | Iannuzzi *et al.* 2001b |
| *PSMA7* | *proteasome (prosome, macropain) subunit, alpha type, 7* | | 13q22prox | 13q22prox | 11 | present study |
| *ADA* | *adenosine deaminase* | | 13q24 | 13q24* | 11 | Iannuzzi *et al.* 2001b |
| *D13S31*  *(IDVGA87)* | *DNA segment* | | 13q24 | 13q22-q23 | 11 | Di Meo *et al.* 2002 |
| *D18S23*  *(IDVGA74)* | *DNA segment* | | 14q12.1 | 18q13 | 9 | Di Meo *et al.* 2002 |
| *EPCDV011* | *DNA segment* | | 14q13 | -------------- | 9 | SheepBase |
| *EPCDV012* | *DNA segment* | | 14q13 | -------------- | 9 | SheepBase |
| *EPCDV019* | *DNA segment* | | 14q13 | -------------- | 9 | SheepBase |
| *CSR00270* | *DNA segment* | | 14q13 |  | 9 | SheepBase |
| *MC1R* | *melanocortin 1 receptor (alpha melanocyte stimulating hormone receptor)* | | 14q13 | 18q13 | 9 | present study |
| *DPEP1* | *dipeptidase 1 (renal)* | | 14q13 | 18q13 | 9 | present study |
| *MT2A* | *metallothionein 2 A* | | 14q15 | 18q14* | 9 | Iannuzzi *et al.* 2001b |
| *GNAO1* | *guanine nucleotide binding protein (G protein), alpha activating activity polypeptide O* | | 14q15 | 18q15 | 9 | present study |
| *D18S17*  *(Haut14)* | *DNA segment* | | 14q22 | 18q21 | 9 | Iannuzzi *et al.* 2003a |
| *D18S22*  *(IDVGA70)* | *DNA segment* | | 14q22 | 18q21 | 9 | Di Meo *et al.* 2002 |
| *RYR1* | *ryanodine receptor 1* *(skeletal)* | | 14q23-q24 | 18q23-q24 | 9 | Iannuzzi *et al.* 2001b |
| *ZNF146* | *zinc finger protein 146* | | 14q24 | 18q24 | 9 | Hayes *et al.* 1996 |
| *LHB* | *luteinizing hormone beta polypeptide* | | 14q24 | 18q24* | 9 | Iannuzzi *et al.* 2001b |
| *GPI* | *glucose phosphate isomerase* | | 14q24 | 18q24 | 9 | Di Meo *et al.* 2003 |
| *D18S21*  *(INRA210)* | *DNA segment* | | 14q24 | 18 | 9 | Iannuzzi *et al.* 2003a |
| *D18S5*  *(INRA063)* | *DNA segment* | | 14q24 | 18 | 9 | Iannuzzi *et al.* 2003a |
| *McM104* | *DNA segment* | | 14q24 | 18q24* | 9 | Iannuzzi *et al.* 2003a |
| *PTGIR* | *prostaglandin I2 (prostacyclin) receptor (IP)* | | 14q24dist | 18q24dist | 9 | present study |
| *EPCDV018* | *DNA segment* | | 15q12 | -------------- | 19 | SheepBase |
| *MMP1* | *matrix metallopeptidase 1 (interstitial collagenase)* | | 15q13 | 15q12* | 19 | Iannuzzi *et al.* 2001b |
| *APOA1* | *apolipoprotein A- I* | | 15q21 | 15q21 | 19 | Iannuzzi *et al.* 2001b |
| *FDX1*  *(AD)* | *Ferredoxin 1* | | 15q21 | 15q14 | 19 | Iannuzzi *et al.* 2001b |
| *HBB* | *hemoglobin,beta* | | 15q23 | 15q22-q27 | 19 | Iannuzzi *et al.* 2001a |
| *D15S16*  *(IDVGA32)* | *DNA segment* | | 15q23 | 15q25 | 19 | Di Meo *et al.* 2002 |
| *D15S13*  *(IDVGA10)* | *DNA segment* | | 15q25 | 15q25 | 19 | Di Meo *et al.* 2002 |
| *FSHB* | *follicle stimulating hormone, beta polypeptide* | | 15q26 | 15q26-q27 | 19 | Di Meo *et al.* 2003 |
| *WT1* | *wilms tumor 1* | | 15q27 | 15q26* | 19 | Iannuzzi *et al.* 2001b |
| *PAX6* | *paired box gene 6 (aniridia, keratitis)* | | 15q27 | 15q27* | 19 | Iannuzzi *et al.* 2001b |
| *BM0848* | *DNA segment* | | 15q29 | 15q29* | 19 | Iannuzzi *et al.* 2003a |
| *MAP1B* | *microtubule-associated protein 1B* | | 16q13.1 | 20q13.1 | 14 | Di Meo *et al.* 2003 |
| *D20S13*  *(BM3517)* | *DNA segment* | | 16q13.1 | 20 | 14 | Iannuzzi *et al.* 2003a |
| *SMN* | *survival of motor neuron* | | 16q13.1 | 20q13.1 | 14 | Iannuzzi *et al.* 2003b |
| *D20S10*  *(TGLA304)* | *DNA segment* | | 16q13.3 | 20 | 14 | Iannuzzi *et al.* 2003a |
| *C9* | *complement component 9* | | 16q17 | 20q17* | 14 | Iannuzzi *et al.* 2001b |
| *NPR3* | *natriuretic peptide receptor C/guanylate cyclase C (atrionatriuretic peptide receptor C)* | | 16q17 | 20q17* | 14 | Iannuzzi *et al.* 2003a |
| *MAF214* | *DNA segment* | | 16q17 | 20 | 14 | SheepBase |
| *PRLR* | *prolactin receptor* | | 16q17 | 20q17 | 14 | Hayes *et al.* 1996 |
| *GHR* | *growth hormone receptor* | | 16q17 | 20q17 | 14 | Sheep Base |
| *SLC6A3* | *solute carrier family 6 (neurotransmitter transporter, dopamine), member 3* | | 16q24 | 20q24 | 14 | present study |
| *LIF* | *leukemia inhibitory factor (cholinergic differentiation factor)* | | 17q13 | 17q12 | 23 | Iannuzzi *et al.* 2001b |
| *FGG* | *fibrinogen, gamma chain* | | 17q13 | 17q13 | 23 | Di Meo *et al.* 2003 |
| *D17S22*  *(OarVH098)* | *DNA segment* | | 17q13 | 17 | 23 | Iannuzzi *et al.* 2003a |
| *D17S21*  *(OarFCB048)* | *DNA segment* | | 17q15 | 17q15* | 23 | Iannuzzi *et al.* 2003a |
| *IL2* | *interleukin 2* | | 17q22 | 17q22dist | 23 | present study |
| *FGF2* | *fibroblast growth factor 2 (basic)* | | 17q23-q25 | 17 | 23 | Pearce *et al.* 1995 |
| *ZNF164* | *zinc finger protein 164* | | 17q24 | 17q24 | 23 | Di Meo *et al.* 2002 |
| *OarCP16* | *DNA segment* | | 17q24 | 17q23* | 23 | Iannuzzi *et al.* 2003a |
| *NOS1* | *nitric oxide synthase 1 (neuronal)* | | 17q24 | 17q25 | 23 | present study |
| *GMBT0011* | *DNA segment* | | 17q23-q26 | -------------- | 23 | SheepBase |
| *EPCDV002* | *DNA segment* | | 17q26 | -------------- | 23 | SheepBase |
| *EPCDV009* | *DNA segment* | | 17q26 | -------------- | 23 | SheepBase |
| *COMT* | *catechol-O-methyltransferase* | | 17q26 | 17q26 | 23 | present study |
| *TGLA122* | *DNA segment* | | 18q12-15 | 21 | 4 | SheepBase |
| *D21S43*  *(IDVGA79)* | *DNA segment* | | 18q15 | 21q14-q15 | 4 | Di Meo *et al.* 2002 |
| *SRCRSP05* | *DNA segment* | | 18q15 | 21q14* | 4 | Iannuzzi *et al.* 2003a |
| *D21S45*  *(ILSTS052)* | *DNA segment* | | 18q15 | 21 | 4 | Iannuzzi *et al.* 2003a |
| *CHRNA7* | *cholinergic receptor, nicotinic, alpha 7* | | 18q17 | 21q17* | 4 | Iannuzzi *et al.* 2001b |
| *D21S12*  *(INRA031)* | *DNA segment* | | 18q22 | 21 | 4 | Iannuzzi *et al.* 2003a |
| *GMBT0016* | *DNA segment* | | 18q23-q24 | -------------- | 4 | SheepBase |
| *CHGA* | *chromogranin A (parathyroid secretory protein 1)* | | 18q24 | 21q23* | 4 | Iannuzzi *et al.* 2001b |
| *PDIA3*  *(GRP58)* | *protein disulfide isomerase family A, member 3* | | 18q24 | 21 | 4 | Iannuzzi *et al.* 2001b |
| *IGH@* | *immunoglobulin heavy locus* | | 18q24 | 21q24 | 4 | Di Meo *et al.* 2003 |
| *SERPINA1*  *(PI)* | *protease inhibitor 1,alpha-1-antitrypsin* | | 18q24 | 21 | 4 | Iannuzzi *et al.* 2001b |
| *BM1303* | *DNA segment* | | 19q12-q13 | -------------- | 12 | SheepBase |
| *DAZL* | *deleted in azoospermia-like* | | 19q13 | -------------- | 12 | [Mandon-Pepin](http://www.ncbi.nlm.nih.gov/entrez/query.fcgi?db=pubmed&cmd=Search&itool=pubmed_Abstract&term="Mandon-Pepin+B"%5BAuthor%5D) *et al.* 2003 |
| *MITF* | *microphthalmia-associated transcription factor* | | 19q22 | 22q22 | 12 | present study |
| *PBXP1* | *pre-B-cell leukemia transcription factor pseudogene 1* | | 19q22 | 22q22 | 12 | present study |
| *RHO* | *rhodopsin (opsin 2, rod pigment) (retinitis pigmentosa 4, autosomal dominant)* | | 19q23-qter | 22q23-q24 | 12 | Ansari *et al.* 1994 |
| *CATHL@* | *cathalecidins* | | 19q24 | 22q24 | 12 | Di Meo *et al.* 2002 |
| *LTF* | *lactotransferrin* | | 19q24 | 22q24 | 12 | Di Meo *et al.* 2003 |
| *HRH1*  *(HistH1)* | *Histamine receptor H1* | | 19q24 | 22q24dist | 12 | Iannuzzi *et al.* 2003a |
| *FHIT* | *fragile histidine triad gene* | | 19q24prox | 22q24prox | 12 | Di Meo *et al.* 2005a |
| *BRD2* | *Bromodomain containing2* | | 20q13 | -------------- | 20 | present study |
| *OLA-DYA* | *major histocompatibility complex, class II, DY alpha (DYA class II MHC leucocyte antigens)* | | 20q13 | 23q13 | 20 | Di Meo *et al.* 2003 |
| *OarCP73* | *DNA segment* | | 20q13 | 23q13* | 20 | Iannuzzi *et al.* 2003a |
| *PPP2R5D* | *Protein phosphatase2, regulatory subunit B(B56),delta isoform* | | 20q15dist | -------------- | 20 | present study |
| *POLR1C* | *Polymerase(RNA) I polypeptide C,30 Kda* | | 20q15dist | -------------- | 20 | present study |
| *TNRC5* | *Trinucleotide repeat containing 5* | | 20q15dist | -------------- | 20 | present study |
| *GSTA1* | *glutathione S-transferase A1* | | 20q22 | 23q15-q21* | 20 | Di Meo *et al.* 2000 |
| *MSH5* | *mutS homolog 5 (E. coli)* | | 20q22 | -------------- | 20 | [Mandon-Pepin](http://www.ncbi.nlm.nih.gov/entrez/query.fcgi?db=pubmed&cmd=Search&itool=pubmed_Abstract&term="Mandon-Pepin+B"%5BAuthor%5D) *et al.* 2003 |
| *OLA-DRB* | *major histocompatibility complex, class I* | | 20q22 | 23q22 | 20 | Di Meo *et al.* 2000 |
| *HSPA1B*  *(HSP70-2)* | *heat shock 70kDa protein 1B* | | 20q22 | 23q22 | 20 | present study |
| *CFB*  *(BF)* | *complement factor B* | | 20q22 | 23q22* | 20 | Di Meo *et al.* 2000 |
| *C4B* | *Complement component 4B* *(Childo blood group)* | | 20q22 | 23q12-q13 | 20 | present study |
| *CDC5L* | *CDC5 cell division cycle 5-like(S.pombe)* | | 20q22 | -------------- | 20 | present study |
| *OLA-DRA2* | *Major histocompatibility complex,class II,DR alpha* | | 20q22 | -------------- | 20 | present study |
| *BAT2* | *HLA-B associated transcript 2* | | 20q22prox |  | 20 | present study |
| *BAT4* | *HLA-B associated transcript 4* | | 20q22prox | -------------- | 20 | present study |
| *EDN1* | *DNA segment* | | 20q24 | 23q25* | 20 | Di Meo *et al.* 2000 |
| *OarHH56* | *DNA segment* | | 20q24 | 23q24* | 20 | Iannuzzi *et al.* 2003a |
| *BMC8012* | *DNA segment* | | 21q13 | 29q15* | 7 | Iannuzzi *et al.* 2003a |
| *OPCML*  *(OCAM)* | *opioid binding protein/cell adhesion molecule-like* | | 21q13 | 29 | 7 | Iannuzzi *et al.* 2000b |
| *D29S35*  *(BMS1112)* | *DNA segment* | | 21q13 | 29 | 7 | present study |
| *LDHA* | *lactate dehydrogenase A* | | 21q22 | 29q22 | 7 | present study |
| *COX8A*  *(COX8)* | *cytochrome c oxidase subunit 8A (ubiquitous)* | | 21q23-q24 | 29q24 | 7 | Iannuzzi *et al.* 2000b |
| *IGF2* | *insulin-like growth factor 2 (somatomedin A)* | | 21q24 | 29q24 | 7 | Di Meo *et al.* 2003 |
| *D29S2*  *(IDVGA7)* | *DNA segment* | | 21q24 | 29q24 | 7 | Di Meo *et al.* 2002 |
| *D29S10*  *(BMC1206)* | *DNA segment* | | 21q24 | 29q24 | 7 | Iannuzzi *et al.* 2003a |
| *FAS*  *(TNFRSF6)* | *Fas (TNF receptor superfamily, member 6)* | | 22q13 | 26q13 | 26 | Di Meo *et al.* 2003 |
| *ACTA2* | *actin, alpha 2, smooth muscle aorta* | | 22q13 | 26q13 | 26 | Iannuzzi *et al.* 2001b |
| *CYP17A1*  *(CYP17)* | *cytochrome P450, family 17, subfamily A, polypeptide 1* | | 22q21-q23 | 26q22-q31 | 26 | SheepBase |
| *DNTT* | *deoxynucleotidyltransferase, terminal* | | 22q21 | 26q21 | 26 | present study |
| *PAX2* | *paired box gene 2* | | 22q21dist. | 26q21 | 26 | present study |
| *D26S14*  *(IDVGA59)* | *DNA segment* | | 22q23 | 26q22 | 26 | Di Meo *et al.* 2002 |
| *MAF0092* | *DNA segment* | | 22q23 | -------------- | 26 | SheepBase |
| *EPCDV020* | *DNA segment* | | 22q23 | -------------- | 26 | SheepBase |
| *OAT* | *ornithine aminotransferase (gyrate atrophy)* | | 22q23dist | 26q23prox | 26 | present study |
| *CYB5A*  *(CYB5)* | *cytochrome b5 type A (microsomal)* | | 23q12 | 24q12 | 28 | present study |
| *MBP* | *myelin basic protein* | 23q11-q12.3 | | 24q12 | 28 | Ansari *et al.* 1994 |
| *DSC1* | *desmocollin 1* | | 23q21 | 24q21 | 28 | Di Meo *et al.* 2003 |
| *DSG2* | *desmoglein 2* | | 23q21 | 24q21-q22 | 28 | present study |
| *ADCYAP1* | *Adenylate cyclase activating polypeptide 1 (pituitary)* | | 23q25 | 24q23 | 28 | Iannuzzi *et al.* 2003a |
| *MAF0035* | *DNA segment* | | 23q25 | -------------- | 28 | SheepBase |
| *ILSTS0043* | *DNA segment* | | 24q12 | -------------- | 8 | SheepBase |
| *HBA1* | *Hemoglobin, alpha 1* | | 24q12 | 25 | 8 | Iannuzzi *et al.* 2000b |
| *EPCDV003* | *DNA segment* | | 24q14 | -------------- | 8 | SheepBase |
| *EPCDV004* | *DNA segment* | | 24q14 | -------------- | 8 | SheepBase |
| *D25S12*  *(IDVGA71)* | *DNA segment* | | 24q14 | 25q12 | 8 | Di Meo *et al.* 2002 |
| *ELN* | *elastin (supravalvular aortic stenosis, Williams-Beuren syndrome)* | | 24q22 | 25q22 | 8 | Di Meo *et al.* 2003 |
| *PRKCB1* | *protein kinase C, beta 1* | | 24q21-q24 | 25 | 8 | SheepBase |
| *D28S12*  *(IDVGA29)* | *DNA segment* | | 25q13 | 28q13 | 29 | Di Meo *et al.* 2002 |
| *TACR2*  *(TAC2R)* | *tachykinin receptor 2* | | 25q14-q19 | 28q14-q23 | 29 | SheepBase |
| *OarVH0072* | *DNA segment* | | 25q14-q15 | -------------- | 29 | SheepBase |
| *CGN1* | *conglutinin 1* | | 25q17 | 28q17 | 29 | Di Meo *et al.* 2003 |
| *D28S10*  *(IDVGA08)* | *DNA segment* | | 25q17 | 28q18-q19 | 29 | Iannuzzi *et al.* 2003a |
| *RBP3* | *Retinol binding protein 3, interstitial* | | 25q19 | 28q18-q19 | 29 | Iannuzzi *et al.* 2003a |
| *RNR5* | *RNA, ribosomal 5* | | 25q19 | -------------- | 29 | SheepBase |
| *MTNR1A* | *melatonin receptor 1A* | | 26q12 | 27q12 | 25 | Messer *et al.* 1997 |
| *DEFB1* | *defensin, beta 1* | | 26q13 | 27q13 | 25 | Di Meo *et al.* 2003 |
| *D27S10*  *(BM6526)* | *DNA segment* | | 26q15 | 27 | 25 | Iannuzzi *et al.* 2003a |
| *F11* | *coagulation factor XI (plasma thromboplastin antecedent)* | | 26q15 | 27q15 | 25 | present study |
| *D27S4*  *(CSSM43)* | *DNA segment* | | 26q17 | 27 | 25 | Iannuzzi *et al.* 2003a |
| *ANK1* | *ankyrin 1, erythrocytic* | | 26q17 | 27q19* ^3^ | 25 | Iannuzzi *et al.* 2000b |
| *PLAT* | *plasminogen activator, tissue* | | 26q19 | 27q18-q19 | 25 | Iannuzzi *et al.* 2000b |
| *OarJMP58* | *DNA segment* | | 26q19 | 27q18*^3^ | 25 | Iannuzzi *et al.* 2003a |
| *D27S6 (HUJI13)* | *DNA segment* | | 26q19 | 27 | 25 | Iannuzzi *et al.* 2003a |
| *DXS113 (DVEPC53)* | *DNA segment* | | Xp11 | Xq41 | X | Iannuzzi *et al.* 2000a |
| *SLC25A6* | *solute carrier family 25 (mitochondrial carrier; adenine nucleotide translocator), member 6* | | Xp12 Yp13 | Xq43 Yp13-p12.2dist | X | Iannuzzi *et al.* 2000a |
| *DXYS3 (TGLA325)* | *DNA segment* | | Xp12 Yp13 | Xq43 Yp13-p12.2dist | X | Iannuzzi *et al.* 2000a |
| *DVEPC102* | *DNA segment* | | Xq11 | Xq35 | X | Iannuzzi *et al.* 2000a |
| *MCM74* | *DNA segment* | | Xq12 | Xq35 | X | SheepBase |
| *DXS30*  *(IDVGA82)* | *DNA segment* | | Xq12 | Xq34 | X | Di Meo *et al.* 2002 |
| *DMD* | *dystrophin (muscular dystrophy, Duchenne and Becker types)* | | Xq12 | Xq33-q34 | X | Iannuzzi *et al.* 2000a |
| *CSRD0241* | *DNA segment* | | Xq12-q21 | -------------- | X | SheepBase |
| *ZFX* | *zinc finger protein, X-linked* | | Xq12 | -------------- | X | SheepBase |
| *ALAS2*  *(ALAS)* | *aminolevulinate, delta-, synthase 2 (sideroblastic/hypochromic anemia)* | | Xq22 | Xq32-q33 | X | Iannuzzi *et al.* 2000a |
| *DXS51*  *(DVEPC027)* | *DNA segment* | | Xq22-q23 | Xq32 | X | SheepBase |
| *PGK1* | *phosphoglycerate kinase 1* | | Xq26 | Xq25-q26 | X | Di Meo *et al.* 2003 |
| *AR* | *androgen receptor (dihydrotestosterone receptor; testicular feminization; spinal and bulbar muscular atrophy; Kennedy disease)* | | Xq26 | Xq25-q26 | X | Iannuzzi *et al.* 2000a |
| *L08239* | *human EST LO8239* | | Xq26 | Xq31 | X | SheepBase |
| *DVEPC132* | *DNA segment* | | Xq31-q32 | Xq21 | X | Iannuzzi *et al.* 2000a |
| *DVEPC137* | *DNA segment* | | Xq32-q34 | Xp11 | X | Iannuzzi *et al.* 2000a |
| *DVEPC076* | *DNA segment* | | Xq32-q34 | Xp11-p12 | X | Iannuzzi *et al.* 2000a |
| *DXS54*  *(DVEPC041)* | *DNA segment* | | Xq32-q34 | Xp11-p12 | X | Iannuzzi *et al.* 2000a |
| *Cos945* | *DNA segment* | | Xq32-q34 | Xq24 | X | SheepBase |
| *F8*  *(F8C)* | *coagulation factor VIII, procoagulant component (hemophilia A)* | | Xq24-q33 | -------------- | X | SheepBase |
| *DXS44*  *(DVEPC014)* | *DNA segment* | | Xq36 | Xp22 | X | Iannuzzi *et al.* 2000a |
| *LAMP2* | *lysosomal-associated membrane protein 2* | | Xq41-q42 | Xp23-p24 | X | Iannuzzi *et al.* 2000a |
| *DXS67*  *(DVEPC065)* | *DNA segment* | | Xq42-q43 | Xp24 | X | Iannuzzi *et al.* 2000a |
| *DIAPH2* | *diaphanous homolog 2 (Drosophila)* | | Xq44 | ---------------- | X | [Mandon-Pepin](http://www.ncbi.nlm.nih.gov/entrez/query.fcgi?db=pubmed&cmd=Search&itool=pubmed_Abstract&term="Mandon-Pepin+B"%5BAuthor%5D) *et al.* 2003 |
| *BTK* | *bruton agammaglobulinemia tyrosine kinase* | | Xq44 | Xq11-q12 | X | Iannuzzi *et al.* 2000a |
| *DVEPC109* | *DNA segment* | | Xq44 | Xq23 | X | SheepBase |
| *PLP1*  *(PLP)* | *proteolipid protein1 (Pelizaeus-Merzbacher disease, spastic paraplegia 2, uncomplicated)* | | Xq44 | Xq11-q12 | X | Iannuzzi *et al.* 2000a |
| *DYZ10*  *(IDVGA50)* | *DNA segment* | | Yp11-q11 | Yp12.1-q12.3 | Y | Di Meo *et al.* 2002 |
| *OPA122* | *DNA segment* | | Yp11 | -------------- | Y | SheepBase |
| *UMN0304* | *DNA segment* | | Yp11- q12prox | Yp12.1-q11 | Y | Di Meo *et al*. 2005b |
| *ZFY* | *zinc finger protein Y-linked* | | Yp12 | Yp12.2 | Y | Di Meo *et al.* 2003 |
| *OPA20* | *DNA segment* | | Yp11 | -------------- | Y | SheepBase |
| *OY1.1* | *DNA segment* | | Yq12 | -------------- | Y | SheepBase |
| *OY11.1* | *DNA segment* | | Yq12 | -------------- | Y | SheepBase |
| *SRY* | *sex determining region Y* | | Yq12prox | Yq12.3 dist | Y | Di Meo *et al.* 2005b |

^1^ Unassigned linkage group.

^2^(<http://www.thearkdb.org/species.html>)

^3^Chromosome location corrected on the basis of the ISCNDB (2001)

**References** (additional table 2)

[Ansari H.A., Pearce P.D., Maher D.W. & Broad T.E](http://www.ncbi.nlm.nih.gov/entrez/query.fcgi?db=pubmed&cmd=Retrieve&dopt=Abstract&list_uids=7536182&query_hl=22&itool=pubmed_docsum). (1994) Regional assignment of conserved reference loci anchors Unassigned linkage and syntenic groups to ovine chromosomes. *Genomics* **24**, 451-5.

Antonacci R., Maccarelli G., Di Meo G.P*. et al.* (2006) Molecular in situ hybridization analysis of sheep and goat BAC clones identifies the transcriptional orientation of T cell receptor gamma genes on chromosome 4 in bovid. *Veterynary Research Communication* (in press).

[Broad T.E., Burkin D.J., Cambridge L.M. *et al.* (1994)](http://www.ncbi.nlm.nih.gov/entrez/query.fcgi?db=pubmed&cmd=Retrieve&dopt=Abstract&list_uids=7919655&query_hl=19&itool=pubmed_docsum) Seven loci on human chromosome 4 map onto sheep chromosome 6: a proposal to restore The original nomenclature of this sheep chromosome. *Mamm Genome* **5**, 429-33.

[Broom M.F. & Zhou C](http://www.ncbi.nlm.nih.gov/entrez/query.fcgi?db=pubmed&cmd=Retrieve&dopt=AbstractPlus&list_uids=11589004&query_hl=3&itool=pubmed_docsum). (2001) Fine mapping of ovine ceroid lipofuscinosis confirms orthology withCLN6. *European Journal Paediatric Neurology*, Suppl A 33-35.

Castiglioni B., Comincini S., Drisaldi B. *et al.* (1998). Comparative mapping of the prion gene (PRNP) locus in cattle, sheep and man with PCR-generated probes. *Mammalian Genome* **9**, 853-5.

Di Meo G.P., Perucatti A., Schibler L. *et al.* (2000) Thirteen type I loci from HSA4q, HSA6p, HSA7q and HSA12q were comparatively FISH-mapped in four river buffalo and sheep chromosomes. *Cytogenetics and Cell Genetics*  **90**, 102-5.

Di Meo G.P., Perucatti A., Incarnato D. *et al.* (2002) Comparative mapping of twenty-eight bovine loci in sheep (*Ovis aries*, 2n=54) and river buffalo (*Bubalus bubalis*, 2n=50) by FISH. *Cytogenetics and Genome Research* **98**, 262-4.

Di Meo G.P., Perucatti A., Gautier M. *et al.* (2003) Chromosome localization of the 31 type I Texas bovine markers in sheep and goat chromosomes by comparative FISH-mapping and R-banding. *Animal Genetics* **34**, 294-6.

Di Meo G.P., Perucatti A., Uboldi C*. et al.* (2005a) Comparative mapping of the fragile histidine triad (FHIT) gene in cattle, river buffalo, sheep and goat by FISH and assignment to BTA22 by RH-mapping: a comparison with HSA3. *Animal Genetics* **36**, 363-4.

Di Meo G.P., Perucatti A., Floriot S. *et al.* (2005b) Chromosome evolution and improved cytogenetic maps of the Y chromosome in cattle, zebu, river buffalo, sheep and goat. *Chromosome Research* **13**, 349-55.

Di Meo G.P., Gallagher D.S., Perucatti A. *et al.* (2006) Mapping of 11 genes by FISH to BTA2, BBU2q, OAR2q and CHI2, and comparison with HSA2q. *Animal Genetics* **37**, 299-300.

[Graphodatsky A.S., Biltueva L.S., Filippov V.A. et al. (1993)](http://www.ncbi.nlm.nih.gov/entrez/query.fcgi?db=pubmed&cmd=Retrieve&dopt=Abstract&list_uids=7679066&query_hl=7&itool=pubmed_docsum) Localization of ESD and A2M genes to sheep chromosome 3 by in situ hybridization. *Cytogenet and Cell Genetics* **62**, 156-8.

[Hediger R., Ansari H.A. & Stranzinger G.F](http://www.ncbi.nlm.nih.gov/entrez/query.fcgi?db=pubmed&cmd=Retrieve&dopt=Abstract&list_uids=1717202&query_hl=9&itool=pubmed_docsum). (1991) Chromosome banding and gene localizations support extensive conservation of chromosome structure between cattle and sheep. *Cytogenetics and Cell Genetics* **57**, 127-34.

[Hayes H., Le Chalony C., Goubin G. *et al.* (1996)](http://www.ncbi.nlm.nih.gov/entrez/query.fcgi?db=pubmed&cmd=Retrieve&dopt=Abstract&list_uids=8641144&query_hl=6&itool=pubmed_docsum) Localization of ZNF164, ZNF146, GGTA1, SOX2, PRLR and EEF2 on homologous cattle, sheep and goat chromosomes by fluorescent in situ hybridization and comparison with the human gene map. *Cytogenetics and Cell Genetics* **72**, 342-6.

Iannuzzi L., Di Meo G.P., Gallagher D.S. *et al.* (1993) Chromosomal localization of omega and trophoblast interferon genes in goat and sheep by fluorescent in situ hybridization. *Journal of Heredity* **84**, 301-4.

Iannuzzi L., Gallagher D.S., Di Meo G.P. *et al.* (1996) High-resolution FISH mapping of beta-defensin genes to river buffalo and sheep chromosomes suggests a chromosome discrepancy in cattle standard karyotypes. *Cytogenetics and Cell Genetics* **75**, 10-3.

Iannuzzi L., Skow L., Di Meo G.P. *et al.* (1997) Comparative FISH-mapping of villin (VIL) gene in river buffalo, sheep and goat chromosomes. *Chromosome Research*  **5**, 199-202.

Iannuzzi L., Palomba R., Di Meo G.P. *et al.* (1998) Comparative FISH-mapping of the prion protein gene (PRNP) on cattle, river buffalo, sheep and goat chromosomes. *Cytogenetics and Cell Genetics* **81**, 202-4.

Iannuzzi L., Gallagher D.S., Di Meo G.P. *et al.* (1999) Comparative FISH-mapping of six expressed gene loci to river buffalo and sheep. *Cytogenetics and Cell Genetics*  **84**, 161-3.

Iannuzzi L., Di Meo G.P., Perucatti A. *et al.* (2000a) Comparative FISH-mapping of bovid X chromosomes reveals homologies and divergences between the subfamilies Bovinae and Caprinae. *Cytogenetics and Cell Genetics* **89**, 171-6.

Iannuzzi L., Di Meo G.P., Perucatti A. *et al.* (2000b) Sixteen type I loci from six chromosomes were comparatively fluorescent in-situ mapped to river buffalo (*Bubalus bubalis*) and sheep (*Ovis aries*) chromosomes. *Chromosome Research* **8**, 447-50.

Iannuzzi L., Gallagher D.S., Di Meo G.P. *et al.* (2001a) Twelve loci from HSA10, HSA11 and HSA20 were comparatively FISH-mapped on river buffalo and sheep chromosomes. *Cytogenetics and Cell Genetics* **93**, 124-6.

Iannuzzi L., Di Meo G.P., Perucatti A. *et al.* (2001b) Comparative FISH-mapping in river buffalo and sheep chromosomes: assignment of forty autosomal type I loci from sixteen human chromosomes. *Cytogenetics and Cell Genetics* **94**, 43-8.

Iannuzzi L., Di Meo G.P., Perucatti A. *et al.* (2003b) Comparative FISH-mapping of the survival of motor neuron gene (SMN) in domestic bovids. *Cytogenetics and Genome Research* **102**, 39-41.

Iannuzzi L., Perucatti A., Di Meo G.P. *et al.* (2003a) Chromosomal localization of sixty autosomal loci in sheep (*Ovis aries*, 2n=54) by fluorescence in situ hybridization and R-banding. *Cytogenetics and Genome Research* **103**, 135-8.

ISCNDB2000 (2001) International System for Chromosome Nomenclature of Domestic Bovids. Di Berardino D., Di Meo G.P., Gallagher D.S., Hayes H., Iannuzzi L. (*co-ordinator*) eds. *Cytogenetics and Cell Genetics*  **92**: 283-99.

[Lopez-Corrales N.L., Sonstegard T.S. & Smith T.P](http://www.ncbi.nlm.nih.gov/entrez/query.fcgi?db=pubmed&cmd=Retrieve&dopt=Abstract&list_uids=9925918&query_hl=23&itool=pubmed_docsum). (1998) Comparative gene mapping: cytogenetic localization of PROC, EN1, ALPI, TNP1, and IL1B in cattle and sheep reveals a conserved rearrangement relative to the human genome. *Cytogenetics and Cell Genetics*  **83**, 35-8.

Mandon-Papin B., Postry-Vaiman A., Vigier B., Piumi F., Cribiu E. & Cotinot C. (2003) Expression profiles and chromosomal localization of genes controllino meiosis and follicular development in the sheep ovary. *Biology of Reproduction* **68** (3); 985-95.

[Massari S., Antonacci R., De Caro F. *et al.* (1997)](http://www.ncbi.nlm.nih.gov/entrez/query.fcgi?db=pubmed&cmd=Retrieve&dopt=AbstractPlus&list_uids=9605848&query_hl=8&itool=pubmed_docsum) Assignment of the TCRA/TCRD locus to sheep chromosome bands 7q1.4-q2.2 by fluorescence in situ hybridization. *Cytogenetics and Cell Genetics* **79**, 193-5.

[Messer L.A](http://www.ncbi.nlm.nih.gov/entrez/query.fcgi?db=pubmed&cmd=Search&itool=pubmed_AbstractPlus&term="Messer+LA"%5BAuthor%5D)., [Wang L](http://www.ncbi.nlm.nih.gov/entrez/query.fcgi?db=pubmed&cmd=Search&itool=pubmed_AbstractPlus&term="Wang+L"%5BAuthor%5D)., [Tuggle C.K](http://www.ncbi.nlm.nih.gov/entrez/query.fcgi?db=pubmed&cmd=Search&itool=pubmed_AbstractPlus&term="Tuggle+CK"%5BAuthor%5D). *et al.* (1997) Mapping of the melatonin receptor 1a (MTNR1A) gene in pigs, sheep, and cattle. *Mammalian Genome* **8**, 368-70.

Pearce P.D., Ansari H.A., Maher D.W. & Broad T.E. (1995) Five regional localizations to the sheep genome: first assignments to chromosome 5 and 12. *Animal Genetics* **26**(3): 171-6.

Perucatti A., Floriot S., Di Meo G.P. *et al.* (2006a) Comparative FISH-mapping of mucin 1, transmembrane (MUC1) among cattle, river buffalo, sheep and goat chromosomes: comparison between bovine chromosome 3 and HSA1. *Cytogenetics and Genome Research* **112**, 103-5.

Perucatti A., Di Meo G.P., Vallinoto M. *et al.* (2006b) FISH-mapping of LEP and SLC26A2 genes in sheep, goat and cattle R-banded chromosomes: comparison between bovine, ovine and caprine chromosome 4 (BTA4/OAR4/CHI4) and human chromosome 7 (HSA7). *Cytogenetics and Genome Research* **115**, 7-9.

[Popescu C.P., Boscher J., Hayes H.C. *et al.* (1995)](http://www.ncbi.nlm.nih.gov/entrez/query.fcgi?db=pubmed&cmd=Retrieve&dopt=AbstractPlus&list_uids=7835086&query_hl=6&itool=pubmed_docsum) Chromosomal localization of the BLV receptor candidate gene in cattle, sheep, and goat. *Cytogenetics and Cell Genetics* **69**, 50-2.

[Vozdova M., Kubickova S., Di Berardino D. & Rubes J](http://www.ncbi.nlm.nih.gov/entrez/query.fcgi?db=pubmed&cmd=Retrieve&dopt=AbstractPlus&list_uids=15008145&query_hl=2&itool=pubmed_docsum). (2003) Assignment of bovine submaxillary mucin (BSM1) gene homologues to bubaline, caprine, and ovine chromosomes by comparative mapping. *Cytogenetics and Genome Research* **103**, 203E.

[Wood N.J., Ansari H.A., Broad T.E. *et al.* (1993)](http://www.ncbi.nlm.nih.gov/entrez/query.fcgi?db=pubmed&cmd=Retrieve&dopt=Abstract&list_uids=8118104&query_hl=15&itool=pubmed_docsum) Regional assignment of the neurotensin locus in sheep. *Mammalian Genome* **4**, 541-3.
